# Real-time volatilomics reveals microbiota and pathogen fingerprints in the honey bee

**DOI:** 10.1128/mbio.01419-26

**Published:** 2026-09-09

**Authors:** Mateusz Fido, Silvia Moriano-Gutierrez, Jiayi Lan, Aurélien Pirat, Léa Zufferey, Elisa Cappio Barazzone, Renato Zenobi, Philip Engel, Emma Slack

**Affiliations:** 1 Department of Chemistry and Applied Biosciences, Swiss Federal Institute of Technology 27219 , Zürich, Switzerland; 2 Department of Fundamental Microbiology, University of Lausanne 27213, Lausanne, Switzerland; 3 Institute of Agrochemistry and Food Technology, National Research Council, Valencia, Spain; 4 Department of Health Sciences and Technology, Swiss Federal Institute of Technology, Zürich, Switzerland; 5 Department of Biology, Swiss Federal Institute of Technology 27219 , Zürich, Switzerland; 6 Sir William Dunn School of Pathology, University of Oxford, Oxford, United Kingdom; 7 Botnar Institute for Immune Engineering, Basel, Switzerland; Universite de Geneve, Geneva, Switzerland

**Keywords:** gut microbiota, honey bee, environmental microbiology, host-pathogen interactions, host-microbiota interactions, pathogenesis, host response, mass spectrometry

## Abstract

Understanding the complex relationship between gut microbiota and their hosts often relies on invasive sampling techniques. Honey bees provide a tractable model for host-microbe studies. Here, we establish single-bee volatilomics using secondary electrospray ionization–high-resolution mass spectrometry (SESI-HRMS) to examine volatile organic compounds released to the air around an individual live honey bee. Specifically, we focused on the primary gut microbiota metabolites present in gnotobiotic bees. Our findings reveal distinct volatilome profiles in honey bees that depend on their gut bacterial colonization state. We cross-validated our findings using an established metabolomics technique, liquid chromatography–high-resolution mass spectrometry (LC-HRMS), to compare and contrast the metabolites detectable with each method. Finally, we assessed the ability of SESI-HRMS to detect colonization with the bee pathogen *Serratia marcescens*. By comparing the volatile signature of this bacterium grown in liquid culture with that of infected honey bee headspace, we identified overlapping compounds, including butane-2,3-diol, that were elevated in infected bees relative to uninfected controls. Non-invasive SESI-HRMS volatilomics, paired with LC-HRMS, therefore, have the potential to identify biomarkers of bee microbiome composition and infection at the level of individual insects. These biomarkers represent practical targets for the development of simple, field-ready diagnostic tools for monitoring pollinator health.

**IMPORTANCE:**

Honey bees are vital to various ecosystems and human agriculture as globally present pollinators. Current declines in bee populations threaten crop productivity and biodiversity and are driven by many factors, including habitat loss, pesticides, and pathogenic infections. The findings presented in this work demonstrate how the analysis of volatile species can precisely detect products of metabolic activity coming from the honey bee and its associated gut microbial community. We were able to demonstrate how high-resolution mass spectrometry can be used to study the host-microbiota relationship on the level of an individual insect. This technique, combined with volatilomics, allowed us to outline a volatile signature of infection in honey bees, which could become a target for in-field beehive disease monitoring.

## INTRODUCTION

In most animals, the gut microbiota is a critical component of host physiology, which influences a wide range of biological functions, including digestion, immune regulation, metabolism, and even behavior (1–6). This symbiotic association is mediated by a diverse array of biochemical interactions, many of which involve the production and exchange of small molecules. Among these, volatile organic compounds (VOCs) represent an important class of metabolites that can serve as indicators of both host physiology and microbial activity. VOCs are produced as metabolic byproducts by both the host and its associated microbiota and provide a unique window into the functional state of an organism. These compounds include alcohols, aldehydes, ketones, organic acids, and short-chain fatty acids (SCFAs), many of which are products of microbial fermentation (1, 7, 8). Beyond their metabolic roles, VOCs function as infochemicals in both intra- and inter-species communication and have been shown to influence host behavior, immune signaling, and disease states (9, 10).

Despite their biological importance, capturing these dynamic metabolic exchanges in a living system has traditionally been challenging. Conventional metabolomics requires invasive sampling techniques, such as tissue dissection, and offers only static snapshots of metabolic activity. Recent advances in volatilomics have paved the way for non-invasive monitoring of metabolic processes in living organisms (11–13). Unlike traditional methods that require disruption of biological systems, volatilomics enables real-time assessment of molecular interactions by detecting VOCs released into an organism’s immediate environment, including low-abundance aerosolized compounds. While this approach has been successfully applied in medical diagnostics (14), food safety (15), and environmental monitoring (16), it remains less explored in social insect host-microbe systems.

Because honey bees provide a tractable social-insect model for host-microbiota studies, their live headspace offers a useful system for testing whether non-invasive VOC measurements can report microbial metabolic activity. Secondary electrospray ionization–high-resolution mass spectrometry (SESI-HRMS) enables the detection of VOCs in real time, without sample preparation or manipulation (17). This approach utilizes an ambient ionization method to detect VOCs present in the headspace of a sample, allowing for non-invasive and dynamic monitoring of host and microbial metabolism. In this method, a gentle flow of a carrier gas directs VOCs toward an electrospray plume, where they are subsequently ionized by collision with charged droplets and separated based on their mass-to-charge ratio in the mass analyzer. This strategy benefits from the very high sensitivity and selectivity of high-resolution mass spectrometry compared to conventional mass spectrometry methods, allowing for the discrimination of VOCs with similar chemical structures.

The Western honey bee (*Apis mellifera*) serves as an excellent model for investigating host-microbiota interactions through volatilomic profiling. It harbors a relatively simple and well-characterized gut microbiota (18–22) and represents an experimentally tractable system that allows for colonizing microbiota-depleted bees with cultured bacteria assembled into defined communities (23, 24). This allows for controlled studies on microbial contributions to host metabolism and volatile production. In honey bees, VOCs play essential roles in chemical communication, foraging, and colony organization (25–27). Recent studies have also shown that the gut microbiota contributes directly to VOC production, including short-chain fatty acids (SCFAs) and other fermentation-derived metabolites that may shape colony odor and host signaling (23, 28–30).

Despite their ecological and economic importance, honey bee populations are globally declining, driven by a combination of stressors, including habitat loss, pesticide exposure, nutritional stress, and pathogenic infections (31, 32). Opportunistic pathogens such as *Serratia marcescens* are emerging as significant contributors to honey bee mortality, particularly in individuals with disturbed or depleted microbiota (33). Current methods for detecting infections or monitoring bee health are limited in resolution, invasiveness, or scalability. Therefore, there is a pressing need for non-invasive, real-time diagnostic tools capable of detecting microbial dysbiosis or pathogen colonization at the individual level.

In this study, we utilize SESI-HRMS to non-invasively characterize the volatilome of living honey bees. By comparing microbiota-depleted bees with those colonized by a synthetic microbial community, we aim to disentangle the contributions of host and microbial metabolism to VOC production and shed light on the critical role of gut microbiota in shaping the chemical signatures of honey bees. This study not only provides new insights into the metabolic interactions between gut microbiota and their insect hosts, contributing to a deeper understanding of microbiome function, but also facilitates the identification of VOC biomarkers linked to microbiota activity. In addition to exploring the role of core gut microbes, this study extends the application of SESI-HRMS to the *in vivo* detection of pathogenic bacteria, specifically *S. marcescens*, an opportunistic pathogen increasingly implicated in honey bee mortality and colony collapse (34). We identify and characterize the VOC signatures associated with *S. marcescens* colonization in the headspace of living individuals. These biomarkers could pave the way for novel, non-invasive diagnostic tools for monitoring honey bee health. Given the global decline in pollinator populations, microbiota-based indicators of bee health could play a crucial role in informing beekeeping strategies and improving colony management practices.

## RESULTS

### Gut microbiota colonization drives distinct volatilome metabolic profiles in honey bees

**Fig 1:**
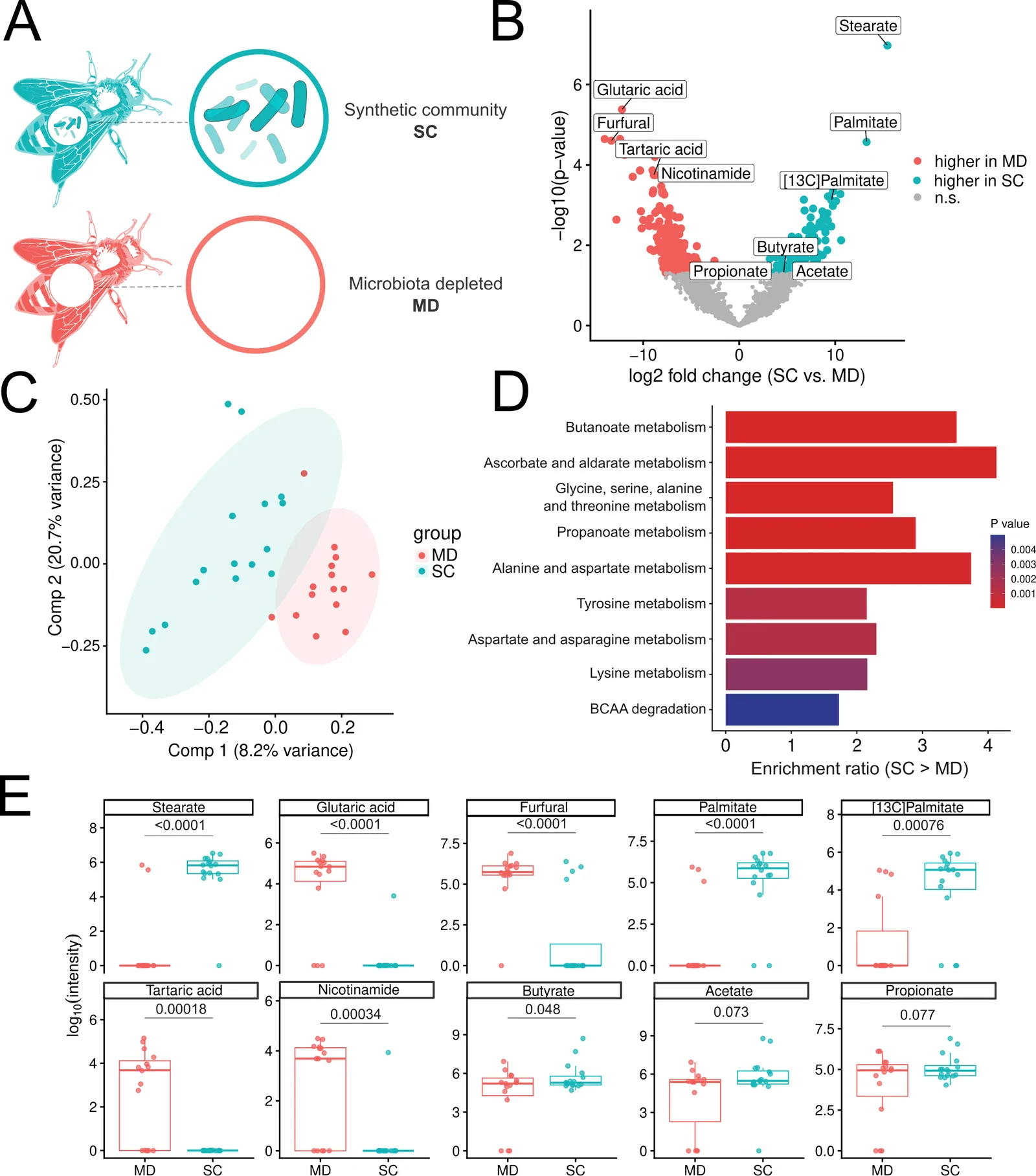
Single-bee measurements of the honey bee headspace volatilome using SESI-HRMS. (**A**) Schematic of the experimental design: microbiota-depleted (MD) and synthetic-community (SC) bees, *n* = 16 per group across four hives and two cages per hive. (**B**) Volcano plot of features shared between SC and MD. Features with *P* <0.05 and |log_2_ fold change| >1 are colored teal (higher in SC) or red (higher in MD); putatively annotated compounds are labeled. (**C**) PLS-DA score plot of SC vs MD bees (2,061 features, group-aware presence filter, leave-one-out cross-validation). (**D**) Pathway enrichment of SC-enriched *m/z* features in the SC > MD direction (Mummichog algorithm, MetaboAnalyst 6.0, *A. mellifera* KEGG library). (**E**) Log_10_ blank-subtracted ion intensities for the seven most differentiating annotated features (stearate, glutaric acid, furfural, palmitate, [^13^C]palmitate, tartaric acid, and nicotinamide) putatively alongside the three microbial-fermentation short-chain fatty acids (butyrate, acetate, and propionate). Welch’s *t*-test with Holm adjustment; adjusted *P*-values are shown above each pair of boxplots. Blank samples correspond to measurements of an empty PMMA tube connected to the carrier gas without a bee inside.

To investigate how gut microbiota shape VOC emissions in honey bees, we compared the headspace profiles of microbiota-depleted (MD) bees and those colonized with a defined synthetic community (SC) using single-bee SESI-HRMS (Fig. 1A). Bees were drawn from four independent hives (1–4). Within each hive, individuals were housed in two separate cages (A and B) to account for potential cage effects, and two bees per cage were measured independently by single-bee SESI-HRMS (16 total single-bee measurements).

Gut microbiota colonization profoundly impacted the global volatile profile of the live animal. Out of 3,570 detected features, 420 showed differential abundance *m/z* values between SC and MD bees, with 259 enriched in MD bees and 161 in SC bees (Fig. 1B). A clear separation between the two groups was observed in the PLS-DA score plot (Fig. 1C), providing more evidence that microbial colonization drives distinct volatile metabolic signatures at the individual level.

Among the top differentiating features, 16 could be putatively annotated through MS/MS library matching (SIRIUS 6.1.1), exact-mass formula assignment, or isotope pattern analysis; the 10 most significant annotated compounds are shown in Fig. 1B and E (Table S2). The most significant were stearate and palmitate (both SC-enriched, *P* < 0.0001), corroborated by detection of the corresponding [¹³C]palmitate isotopolog (*P* = 7.6 × 10^−4^). Several metabolites enriched in MD bees pointed to an accumulation of host-derived organic acids in the absence of microbial metabolism, including glutaric acid (*P* = 4.2 × 10^−6^), tartaric acid (*P* = 1.8 × 10^−4^), and nicotinamide (*P* = 3.4 × 10^−4^), together with furfural (*P* = 2.5 × 10^−5^), which strongly co-varies with glutaric acid (*r* = 0.75) and therefore likely stems from fragmentation of co-occurring dicarboxylic acids in the ion source of the mass spectrometer.

We included the three short-chain fatty acids butyrate, acetate, and propionate in the right-hand boxplots of Fig. 1E because of biological knowledge of microbial fermentation in the bee gut, together with the butanoate and propanoate pathway enrichment in Fig. 1D, predicts that they should differ between SC and MD bees. In our single-bee headspace data, butyrate reaches the *P* < 0.05 significance threshold after Holm adjustment (*P* = 0.048), whereas acetate and propionate show SC >MD trends without crossing this threshold (*P* = 0.073 and *P* = 0.077, respectively), and the per-bee distributions overlap substantially between groups. Acetate, propionate, butyrate, and valerate were nevertheless consistently detected above background levels in both MD and SC bees (*P* < 0.0001 vs blanks; Supplemental material), whereas formate and caproate displayed high intensities in the blanks, likely due to ambient contamination, and could not be reliably differentiated at the single-bee level.

Pathway enrichment of SC-elevated *m/z* values (Mummichog algorithm, MetaboAnalyst 6.0, *A. mellifera* KEGG library) highlighted short-chain fatty acid metabolism (butanoate and propanoate), ascorbate and aldarate metabolism, and several amino-acid pathways as the most enriched in SC relative to MD bees (Fig. 1D).

Together, these results demonstrate that gut microbiota colonization profoundly shapes the honey bee volatilome, modulating short-chain fatty acid metabolism (butanoate and propanoate), amino-acid metabolism (BCAA, glycine/serine/alanine/threonine, tyrosine, and alanine/aspartate), and the abundance of saturated long-chain fatty acids (palmitate and stearate), a signature consistent with microbial fermentation and amino-acid catabolism within the gut. This is in agreement with predictive functional analysis of the microbial communities found via 16S rRNA gene sequencing of the MD and SC guts (Fig. S4; Supplemental material).

### SESI-HRMS of live bees and LC-HRMS of bee guts cover distinct but complementary areas of the honey bee metabolome

**Fig 2:**
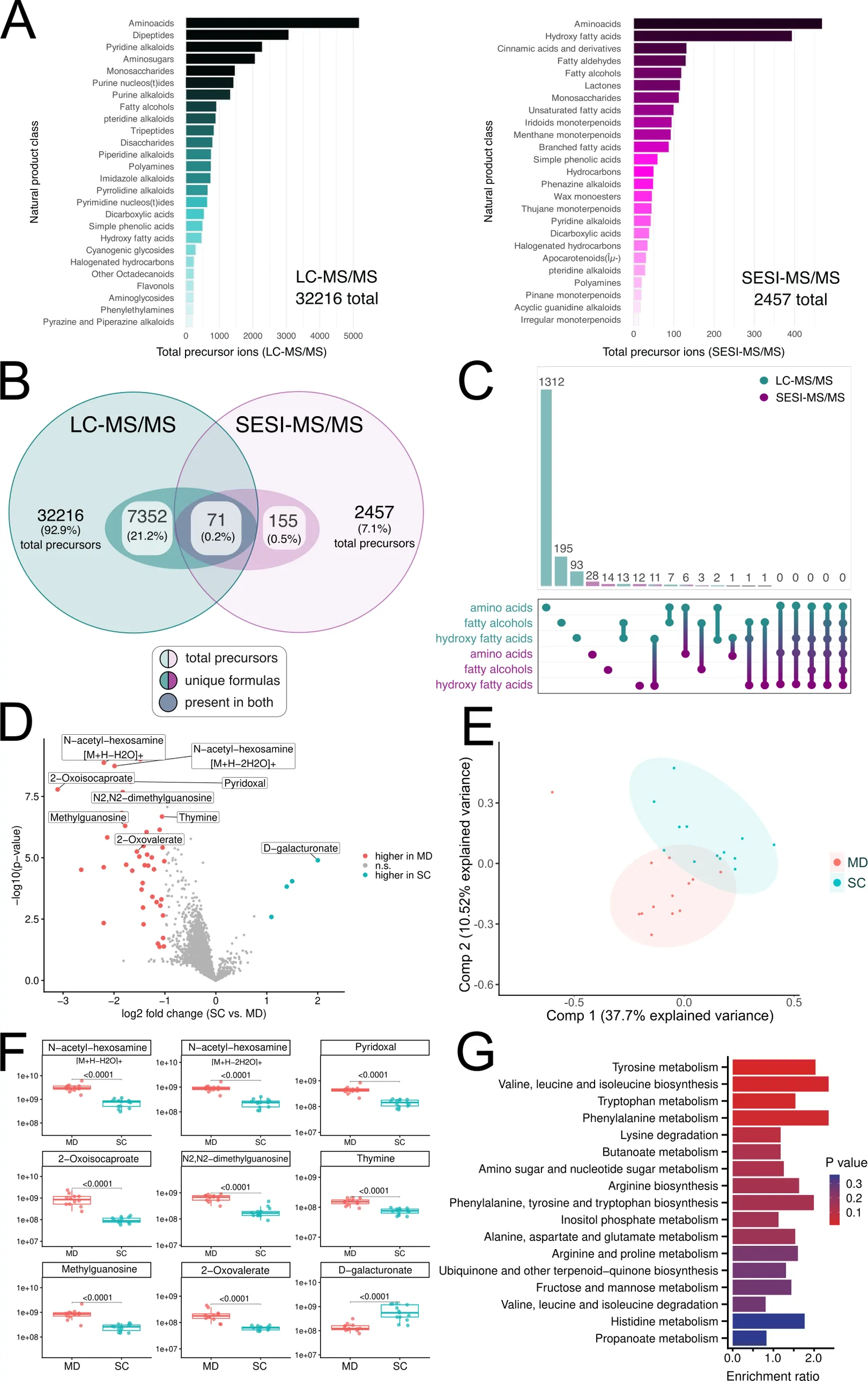
LC-HRMS comparison of the impact of gut microbiota on the metabolomic profiles of honey bee guts. (**A**) Distribution of the most abundant natural product classes (NPCs) of the molecular formulas identified with LC-MS/MS (left) and SESI-MS/MS (right). (**B**) Venn diagram showing the shared distribution of all the detected and putatively identified precursor ions in LC-MS/MS and SESI-MS/MS. (**C**) Upset plot showing the overlap of compounds classified as amino acids, fatty alcohols, and hydroxy fatty acids between LC-MS/MS and SESI-MS/MS. (**D**) Volcano plot showing the differential abundances of features found in MD and SC bees via LC-HRMS. Features with *P* < 0.05 and absolute fold change >2 are highlighted in blue (higher in SC) and red (higher in MD). (**E**) PLS-DA plot showing the distribution of samples belonging to the gut microbiota-depleted (MD) and synthetic community (SC) groups via LC-HRMS. (**F**) Boxplots showing the MS intensity of the nine MS/MS-annotated compounds with the lowest Welch’s *t*-test *P*-values between SC and MD bee guts by LC-HRMS. Individual replicates are overlaid on the boxplots; *P*-values above each panel are the raw Welch’s *t*-test values. (**G**) Enrichment analysis of the differentially abundant MS/MS-identified compounds from panel **F** (Mummichog module of MetaboAnalyst 6.0, *A. mellifera* KEGG library).

To evaluate how headspace metabolomics by SESI-HRMS relates to conventional liquid chromatography-based metabolomics, we analyzed the water-extracted fraction of homogenized gut tissue of the same bees analyzed by SESI-HRMS (Fig. 1), using LC-HRMS (Fig. 2A). This paired analysis enabled a direct comparison of chemical coverage, compound annotation, and microbiota-associated metabolic shifts captured by each technique and body site.

Using the CANOPUS classification algorithm (a deep neural network tool for systematic classification of unknown metabolites) (35), we found that amino acids were the most abundant natural product class detected in both LC-MS/MS and SESI-MS/MS (Fig. 2A). However, the two platforms captured largely distinct molecular populations. LC-MS/MS detected a total of 32,216 precursor ions, representing 92.9% of all features, whereas SESI-MS/MS detected 2,457 precursor ions (7.1%). Only 71 molecular formulas (0.2%) were shared between the two data sets (Fig. 2B). Among these, only six formulas corresponding to amino acids overlapped between the methods (Fig. 2C), confirming that SESI-MS/MS and LC-MS/MS probe separate regions of the honey bee metabolome.

The remaining natural product classes were largely associated with the phase of the sample. LC-MS/MS was dominated by amino acids, peptides, alkaloids, and sugars, whereas the most abundant molecules in SESI-MS/MS constituted hydroxy fatty acids, cinnamic acids (plant-derived aromatic compounds present in honey, pollen, and bee propolis), and fatty alcohols and aldehydes (Supplemental material).

Overall, we found a much greater number of precursor ions in LC-MS/MS than SESI-MS/MS. Out of these, 7,352 (21.2%) were attributable to unique molecular formulas, which resulted in many more hits being identifiable with MS/MS (Supplemental material).

Comparison of the shared *m/z* features found in the gut of MD and SC bees by LC-HRMS revealed pronounced group-specific metabolic differences (Fig. 2D through F). In total, most significantly altered metabolites were elevated in MD bees, paralleling the pattern observed in SESI-HRMS (Fig. 2D). PLS-DA demonstrated clear separation between MD and SC samples (Fig. 2E), indicating that gut microbial colonization strongly influences gut metabolite composition. Predominantly, the compounds upregulated in MD bees were nucleosides (guanosine and adenosine), amino sugars, and keto acids, which accumulate during incomplete breakdown of branched-chain amino acids (BCAAs). Amino acids implicated in previous work on gnotobiotic honey bees (phenylalanine, glutamine, and lysine) showed modest enrichment in SC bees but did not reach statistical significance and are therefore excluded from the Fig. 2F boxplot panel, which displays the nine MS/MS-annotated features with the lowest Welch’s *t*-test *P*-values.

The most discriminant compounds identified by MS/MS included the vitamin B₆ cofactor pyridoxal, methylated nucleosides (methylguanosine and N²,N²-dimethylguanosine), the nucleobase thymine, and intermediates of branched-chain amino acid (BCAA) catabolism such as 2-oxo-isocaproate and 2-oxovalerate (Fig. 2F). The most statistically significant feature, annotated as N-acetyl-hexosamine, appeared as two distinct ions differing by neutral water losses. While these *m/z* values could represent the same molecular species, it is metabolically more likely that several amino sugar epimers (e.g., N-acetyl-glucosamine and N-acetyl-galactosamine), indistinguishable without targeted follow-up such as derivatization or selected high-resolution ion mobility.

To assess the metabolic impact of the differentially abundant metabolites, we applied the same enrichment analysis used for SESI-HRMS (Fig. 2G). LC-HRMS data revealed a strong representation of BCAA biosynthesis and degradation; pyrimidine and purine metabolism; phenylalanine, tyrosine, and tryptophan metabolism; and vitamin B₆ metabolism, confirming the earlier findings. Consistent with the SESI-HRMS data set, butanoate and glyoxylate/dicarboxylate metabolism also appeared significantly enriched, supporting the notion that gut microbiota modulate host amino acid and fatty acid biosynthesis pathways across both volatile and non-volatile metabolomic spaces.

Collectively, these results show that SESI-HRMS on live bees and LC-HRMS on isolated intestine provide complementary insights into the honey bee metabolome, with LC-HRMS capturing hydrophilic gut metabolites linked to amino sugar, amino acid, and nucleotide metabolism, and SESI-HRMS profiling the corresponding volatile fermentation products and fatty acids emitted by the host.

### Butane-2,3-diol is a volatile metabolic signature of *S. marcescens* detectable in the headspace of infected honey bees

**Fig 3:**
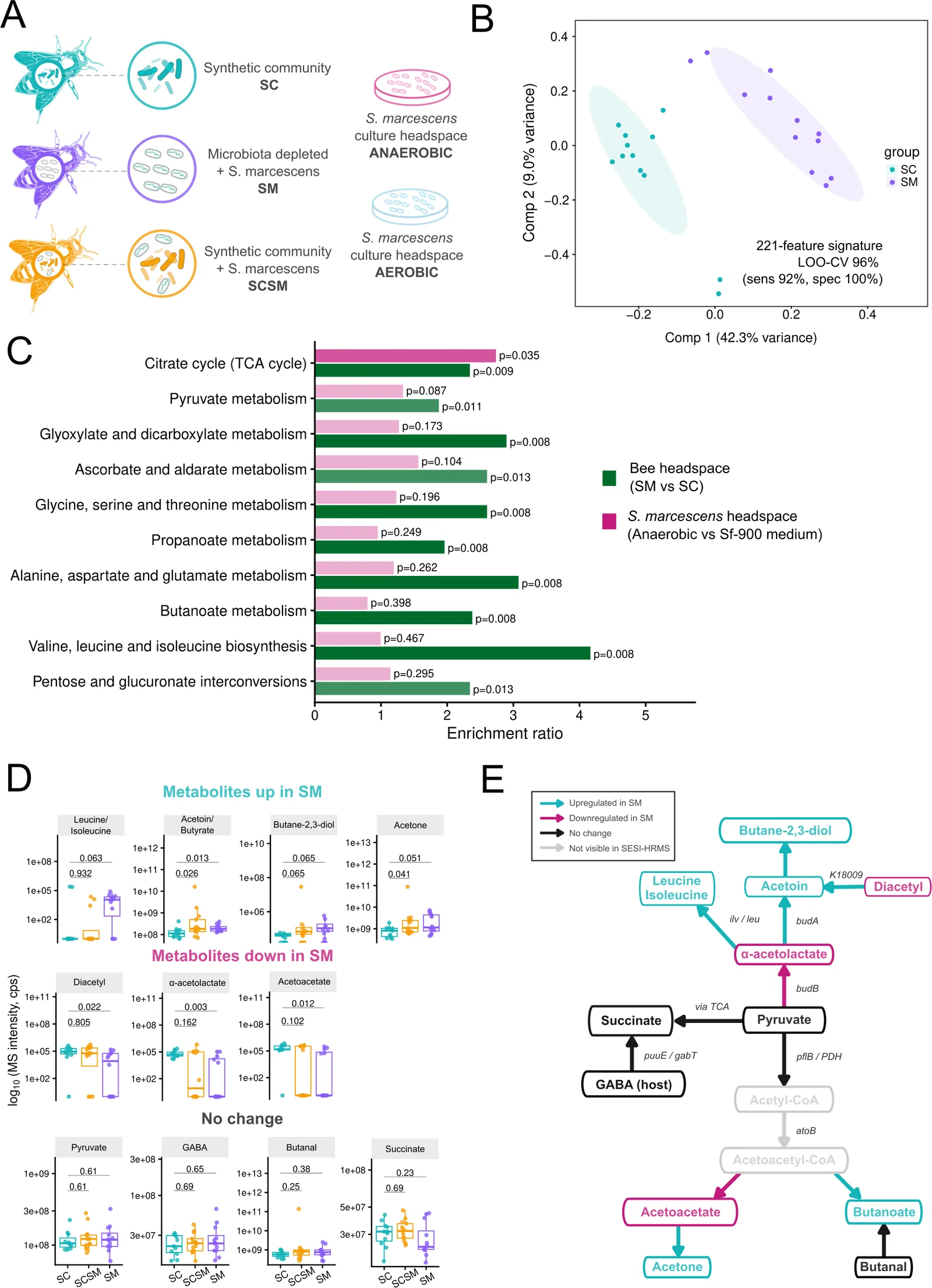
*S. marcescens* colonization accumulates pyruvate fermentation products in the honey bee headspace. (**A**) Schematic of the experimental design. SC, bees colonized with a synthetic community of bacteria; SM, bees monocolonized with *S. marcescens*; SCSM, bees co-colonized with both. Four biological replicates and three individual bees per replicate. *S. marcescens* cultures were grown in Sf-900 liquid medium under fully aerobic and fully anaerobic conditions (three biological replicates each), with their headspace analyzed by SESI-HRMS in parallel. (**B**) PLS-DA of bee headspace samples projected onto the 221-feature *S. marcescens* signature, validated by a LOO-CV classifier. (**C**) Combined pathway enrichment of (i) SM-elevated features in the bee headspace (green bars, *A. mellifera* KEGG library) and (ii) anaerobic-vs-Sf-900-medium contrast in *S. marcescens* liquid culture (magenta bars, *S. marcescens* KEGG library), both computed with the Mummichog module of MetaboAnalyst 6.0 in mixed ion mode at 5 ppm. Bars show enrichment ratios; color intensity encodes the Mummichog Gamma *P*-value. (**D**) Signature boxplots of 11 *m*/*z* features implicated in the proposed mechanism. Wilcoxon rank-sum with Holm correction per panel and pairwise *P*-values are shown above each comparison (SCSM-vs-SC and SM-vs-SC brackets). (**E**) Proposed mechanism by which *S. marcescens* metabolism influences the host volatilome; gray nodes indicate non-volatile metabolites not detected by SESI-HRMS.

The gram-negative species *S. marcescens* is an opportunistic pathogen of bees that causes elevated mortality in microbiota-depleted individuals but is largely suppressed in bees harboring a normal gut community (7, 33, 34)*. Serratia* cultures are known to produce many volatile organic compounds, including various ketones, alcohols, and compounds involved in butanoate metabolism (36–40). We investigated the volatile signatures of *S. marcescens* by measuring isolated cultures grown under fully aerobic and anaerobic conditions and then compared them to the headspace of honey bees colonized with either a defined synthetic community (SC), *S. marcescens* alone (SM), or both together (SCSM) (Fig. 3A). Each condition included four biological replicates with three bees per replicate (from four different hives), measured individually 5 days post-inoculation.

We profiled the headspace of individual bees from the three colonization groups (SC, SM, and SCSM; *n* = 12 bees per group, four biological replicates with three bees each) in parallel with the headspace of *S. marcescens* liquid cultures grown in Sf-900 medium under fully aerobic and fully anaerobic conditions (three biological replicates per condition) (Fig. 3A). Of the 2,969 *m*/*z* features shared between the three bee groups, 153 were elevated in SM bees and 87 in SC bees using raw *P* < 0.05 and fold change >2. To test whether the global headspace profile discriminates infection status, we trained a two-class PLS-DA (SM vs SC) on the blank-subtracted, group-aware-filtered feature matrix, which reached 79.2% leave-one-out cross-validation accuracy (LOO-CV, 10/12 SC, and 9/12 SM correctly classified). Restricting the input to a 221-feature signature (volcano-significant ∩ VIP >1 from the same contrast) raised LOO-CV accuracy to 96% (sensitivity 92%, specificity 100%, reference group = SC; Fig. 3B). SCSM samples, projected onto the same axes for visualization, fall between the SC and SM centroids, consistent with partial *S. marcescens* suppression by the native community through colonization resistance; the three-class (SC/SCSM/SM) score plot (Fig. S11 and S4) shows the same qualitative pattern.

The citrate cycle (TCA cycle) was the clearest shared signal and was significant at *P* < 0.05 in both the bee-headspace and culture analyses (bee *P* = 0.009; *S. marcescens P* = 0.035). Several additional pathways followed behind this signal in the merged pathway view, including pyruvate metabolism; glyoxylate and dicarboxylate metabolism; ascorbate and aldarate metabolism; glycine/serine/threonine metabolism; propanoate metabolism; alanine, aspartate, and glutamate metabolism; butanoate metabolism; valine/leucine/isoleucine biosynthesis; and pentose and glucuronate interconversions, although these were weaker in the culture contrast. Together, this pathway set converges on α-acetolactate, the branch-point metabolite linking pyruvate fermentation, butanoate metabolism, and branched-chain amino-acid (BCAA) biosynthesis in *S. marcescens*. This shared node is the anchor for the compound-level analysis in Fig. 3D and E.

To resolve this signature into individual compounds, we profiled 11 *m/z* features around α-acetolactate in the SM-vs-SC contrast (Wilcoxon rank-sum with Holm correction, reference group = SC; Fig. 3D). Four compounds accumulate in SM bees (Fig. 3D, row 1): leucine/isoleucine (isobaric, [M + H]^+^ 132.102, *P* = 1.3 × 10^−2^), acetoin/butyrate (isobaric, [M + H]^+^ 89.060, *P* = 6.0 × 10^−3^), butane-2,3-diol ([M + H]^+^ 91.076, *P* = 6.0 × 10^−2^), and acetone ([M + H]^+^ 59.050, *P* = 7.6 × 10^−2^). Three compounds are depleted in SM bees (Fig. 3D, row 2): diacetyl (*P* = 2.7 × 10^−2^), α-acetolactate (*P* = 1.2 × 10^−3^), and acetoacetate (*P* = 1.2 × 10^−2^). Four compounds do not change significantly (Fig. 3D, row 3): pyruvate (*P* = 0.61), GABA (*P* = 0.65), butanal (*P* = 0.38), and succinate (*P* = 0.23). We note that acetoin and butanoate (butyrate) are isobaric at *m/z* 89.060 and yield overlapping pseudomolecular ions; only butanoate can be synthesized by the honey bee host, whereas acetoin is a typical *S. marcescens* fermentation product; hence, the elevated signal at this *m/z* in SM bees most likely reflects a mixed contribution dominated by *S. marcescens*-derived acetoin. Butane-2,3-diol reaches only borderline significance (*P* ≈ 0.06) and is also detectable at a background level in some SC individuals and the sterile Sf-900 medium; it is therefore one member of a multi-compound fermentation signature, rather than a reliable standalone marker. Beyond these 11 pathway-mapped compounds, the untargeted feature matrix also contains two additional *S. marcescens*-characteristic markers elevated in SM bees: tyramine ([M + H]^+^ 138.091, *P* = 2.5 × 10^−2^), the product of bacterial tyrosine decarboxylation documented in Enterobacterales, and 3-methylbutyrate (isovalerate, SIRIUS-annotated C_5_H_10_O_2_ at [M + H]^+^ 103.075, *P* = 2.1 × 10^−2^), a branched-chain fatty acid produced by leucine catabolism; full feature tables are provided in the Supplemental material.

To place these 11 compounds onto the biochemistry that generates them, we mapped their direction of change onto the KEGG *S. marcescens* butanoate/BCAA metabolism pathway (smar00650; Fig. 3E). α-acetolactate, the immediate product of α-acetolactate synthase budB, is depleted in SM bees, consistent with its onward consumption by α-acetolactate decarboxylase budA toward acetoin and, via the ilv/leu branch, toward the BCAA end-products leucine/isoleucine; both budA and budB are biochemically characterized in *S. marcescens*. Acetoin is further reduced to butane-2,3-diol by the K18009 diacetyl/2,3-butanediol reductase, and both products accumulate in SM bees; diacetyl, the parallel substrate of the same reductase, is depleted. On the β-keto-acid side, acetoacetate is depleted, while acetone accumulates, consistent with non-enzymatic β-decarboxylation. The pyruvate “hub” itself remains stable (*P* = 0.61), indicating a high-throughput flux state rather than substrate accumulation. Succinate (TCA) and butanal are likewise unchanged, as is GABA, consistent with the *S. marcescens* KEGG annotation lacking glutamate decarboxylase (gadB/K01580), so that any detected GABA must be host-derived rather than bacterially produced. Acetyl-CoA and acetoacetyl-CoA (gray nodes in Fig. 3E) are non-volatile and not detected by SESI-HRMS.

Finally, to assess the bacterial origin of the SM-bee-accumulated compounds, we measured the headspace of the sterile Sf-900 medium used to grow *S. marcescens* (single biological replicate; Fig. S8). The medium blank showed negligible signals for acetoin, butane-2,3-diol, and acetone, consistent with their bacterial origin in the culture; because only one medium blank was measured, we cannot definitively attribute every individual volatile in the *S. marcescens* culture headspace exclusively to bacterial metabolism, and interpret the culture data cautiously. Aerobic and anaerobic *S. marcescens* cultures produced broadly similar profiles for all butanoate-pathway targets (all pairwise *P* > 0.09; Fig. S6 and S8), and we accordingly describe the culture VOC profile without distinguishing between the two conditions.

Taken together, Fig. 3B through E show that *S. marcescens* colonization of the honey bee gut coordinates a reproducible reprogramming of the headspace volatilome centered on α-acetolactate: substrates of the pyruvate-fermentation branch (α-acetolactate, diacetyl, and acetoacetate) are consumed, fermentation products (acetoin, butane-2,3-diol, and acetone) and the BCAA end-product (leucine/isoleucine) accumulate, and two orthogonal *Serratia* markers from aromatic amino acid and BCAA degradation pathways (tyramine, 3-methylbutyrate) corroborate the signal, captured in real time from the bee headspace.

## DISCUSSION

This study demonstrates that volatile metabolite profiling using SESI-HRMS provides a sensitive and non-invasive approach for characterizing host-microbe metabolic interactions in the honey bee. By integrating single-bee volatilomics with LC-HRMS analysis of gut extracts, we uncovered distinct chemical signatures associated with (i) gut microbiota colonization, (ii) microbiota depletion, and (iii) bacterial infection.

Volatilomics offers a convenient and non-invasive sampling and analysis strategy, compared to traditional experimental interventions in metabolomics. It aims to minimize the necessary handling and stress incurred on the animal and has been successfully used in metabolomic studies in many other model organisms, from bacteria to humans (11, 12, 23, 41). In this work, we used the Western honey bee as a gnotobiotic model with a relatively well-defined and simple gut microbial composition to examine the metabolic differences detectable in their volatilome using SESI-HRMS. SESI-HRMS has proven particularly effective in diverse biological systems, from bacterial cultures to mammalian breath analysis, and here we demonstrate applicability to an insect host.

Our initial examination of the headspace of bees shows that the gut microbiota profoundly alters the host volatilome (Fig. 1). Bees colonized with a defined SC displayed distinct volatile signatures compared to MD bees, particularly within short-chain fatty acid fermentation (butanoate and propanoate), amino-acid metabolism (BCAA degradation, glycine/serine/alanine/threonine, tyrosine, and alanine/aspartate), and the accumulation of saturated long-chain fatty acids. Fatty acids play a number of very important roles in bee health, including changes in learning and cognition, energy metabolism, cellular membrane structure, environmental adaptation, and immune responses (41–44). Among the volatiles enriched in colonized bees, stearate and palmitate dominated the profile, both key precursors for lipid biosynthesis and royal jelly constituents (45, 46), and are the most abundant FAs in honey bees overall (45–47). We additionally observed higher on-average concentrations of the SCFAs acetate, propionate, and butyrate in SC bees, consistent with the butanoate and propanoate pathway enrichment in Fig. 1D, although at the single-bee compound level, only butyrate reached significance (*P* = 0.048), and acetate/propionate remained above the *P* < 0.05 threshold (*P* = 0.073, *P* = 0.077) (8, 48).

Our complementary LC-HRMS analysis confirmed extensive metabolic remodeling associated with microbiota colonization. MD exhibited over 13 times more upregulated ions than SC bees (3,728; Fig. 2D), consistent with an accumulation of dietary material that would normally be metabolized by the gut microbiota. Previous work showed that the honey bee gut community, particularly *Gilliamella apicola*, degrades pollen-derived polysaccharides such as pectin and is associated with elevated uronic-acid metabolites in colonized bees (48–50). Consistent with this, the most strongly SC-enriched LC-HRMS feature matched a C6 sugar acid signal annotated as galacturonate/glucuronate, alongside amino acid-related metabolites (Fig. 2D through G). Although the MS/MS signature is consistent with galacturonate, recent work suggests that signals previously attributed to galacturonate may instead reflect host-derived glucuronate and downstream ascorbate metabolism. In agreement with this interpretation, we observed significantly elevated L-threonate, an ascorbate degradation product, in SC bees.

We also found a high abundance of nucleosides and nucleobases in the guts of MD bees, particularly methylated guanosines and thymine (Fig. 2F), along with adenosine. Adenosine is associated with neurotransmission and stress responses in *A. mellifera*, and higher levels of adenosine receptors were found in the bodies of bees infected with the common deformed wing virus, whereas lower levels were found in their brains (51). This suggests that the systemic response to the lack of beneficial gut community resembles the one caused by an infection. Thus, gut dysbiosis provokes stress and immune pathways resembling pathogenic challenge, a pattern conserved across insects and vertebrates.

When comparing the compound identification capabilities of SESI-MS/MS and LC-MS/MS, we found a very high number of successful high-confidence MS/MS matches in LC-MS/MS (Fig. 2A). Feature annotation is one of the biggest challenges in untargeted *-omics* techniques, which makes preseparation and MS capabilities a major advantage in mass spectrometry-based workflows. Direct infusion (DI) techniques, while offering high throughput and real-time analysis capability, do not have this advantage. This is the likely reason for 10 times lower MS/MS identification rate in SESI-MS (Fig. 2A through C), and a known problem in DI-MS.

While we found amino acids to be the most highly represented group in both LC-MS/MS and SESI-MS/MS, we found little overlap between the actual formulas and structures (Fig. 2C). Amino acids generally have extremely low vapor pressures and are not considered volatile (e.g., at 25°C glycine has a vapor pressure of only 1.73 × 10^−8^ kPa compared to 30.6 kPa for acetone, a very well-ionizable standard analyte in SESI) (52, 53). Indeed, when manually examining the actual compound assignments (Supplemental material), it becomes apparent that the amino acids visible in SESI are rarely alpha amino acids: instead, they are VOCs like γ-aminobutyric acid (GABA) and aminocaproic acid, which contribute to this natural product assignment. However, it is challenging to carry out LC-HRMS and SESI-HRMS on exactly the same samples. SESI-HRMS measures compounds in the gas phase at ambient concentrations, which are orders of magnitude lower than in the tissue extracts used for LC-HRMS. Therefore, we cannot conclusively link this discrepancy to the volatility of these compounds, but it remains the strongest working hypothesis for the observed differences.

As a final proof of concept for the applicability of volatile analysis to monitoring honey bee metabolic responses and health, we found bees colonized with *S. marcescens* to have a distinct volatile profile from healthy controls, separable by PLS-DA at 96% leave-one-out cross-validation accuracy (Fig. 3B). Mummichog pathway enrichment of SM features against the *A. mellifera* KEGG library and of *S. marcescens* liquid culture features against the *S. marcescens* KEGG library identified 10 overlapping pathways that converge on α-acetolactate (Fig. 3C), the metabolic node shared by pyruvate fermentation, butanoate metabolism, and branched-chain amino-acid biosynthesis in *S. marcescens*. At the compound level, this shared node resolves into a coherent pattern (Fig. 3D and E): fermentation substrates (α-acetolactate, diacetyl, and acetoacetate) are depleted in SM bees, while downstream fermentation products (acetoin/butyrate, butane-2,3-diol, and acetone) and the BCAA end-product leucine/isoleucine accumulate. Since SM bees lack a healthy gut microbial community, they would be expected to show low levels of butyrate and related butanoate-pathway intermediates; the observed enrichment therefore indicates that *Serratia* itself actively produces these metabolites, rather than this being a host response to infection. The honey bee host lacks the enzymatic machinery of budA/budB and of the K18009 diacetyl/2,3-butanediol reductase and therefore cannot produce acetoin or butane-2,3-diol; hence, their detection in SM bees is evidence consistent with *S. marcescens* fermentation within the host, emitted through the volatile headspace and captured by SESI-HRMS in real time and non-invasively. The intermediate volatilome of SCSM bees (Fig. S11) is consistent with partial suppression of *S. marcescens* by the native microbiota through colonization resistance.

Butane-2,3-diol is moderately volatile (182°C boiling point, 0.03 kPa under standard conditions) and poorly ionizable via SESI, yielding low absolute MS intensity. However, it has few molecular interferences beyond its own isomers; other common compounds with this molecular formula are industrial solvents unlikely to be found in the hive. Our data indicate that butane-2,3-diol alone is not sufficiently reliable as a standalone biomarker of *S. marcescens* infection: in the bee headspace, it reaches only borderline significance (*P* ≈ 0.06), is detectable in some SC individuals, and the sterile Sf-900 medium blank was measured as a single biological replicate (Fig. S8); hence, we do not rely on this compound alone. Nonetheless, butane-2,3-diol is one of four pyruvate-fermentation products that accumulate coherently in SM bees, together with acetoin/butyrate, acetone, and the BCAA end-product leucine/isoleucine (Fig. 3D, row 1). The same flux is corroborated by the parallel depletion of three pathway substrates (α-acetolactate, diacetyl, and acetoacetate; Fig. 3D, row 2), by the shared pathway enrichment between bee headspace and *S. marcescens* liquid culture (Fig. 3C), and by two orthogonal markers from aromatic amino acid and BCAA degradation pathways (tyramine, 3-methylbutyrate; Supplemental material). This combinatorial fingerprint provides robust evidence of active *S. marcescens* metabolism within the host. Further research is required to evaluate the sensitivity and specificity of this multi-compound approach in the field and to explore the potential of SESI-HRMS to differentiate economically relevant bee pathogens at early stages of infection.

Methodologically, combining SESI-HRMS with LC-HRMS provides a multidimensional view of host-microbe metabolism, spanning both volatile and non-volatile molecular spaces. Looking forward, the miniaturization of SESI interfaces and the development of portable or field-deployable VOC sensors could enable real-time, hive-side monitoring of honey bee health. Chemical markers such as acetoin or butane-2,3-diol could be integrated into biosensor systems to detect early signs of dysbiosis, nutritional stress, or infection, offering practical applications for pollinator management and conservation. More broadly, extending volatilomics to other insects and animal models may illuminate general principles of host-microbe metabolic communication and reveal conserved volatile mediators of symbiosis, immunity, and disease.

**Fig 4:**
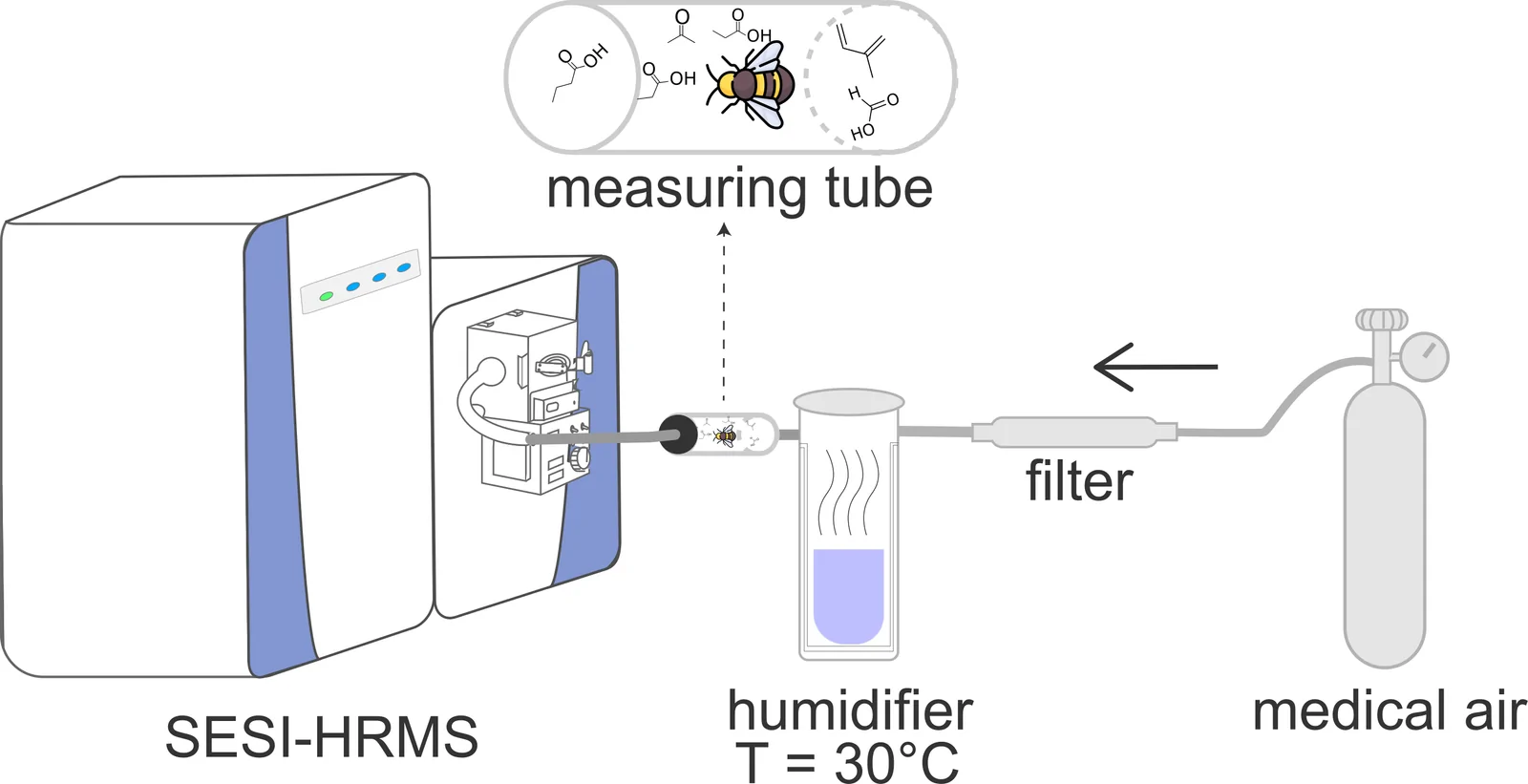
Schematic representation of the in-house headspace sampling system used for measuring volatiles released from honey bees.

The limitations of this work include honey bees analyzed from one season only, as well as the lack of controls explicitly varying diet prior to analysis. Bees were provided sterile irradiated pollen after emergence, but pollen was removed 24 h before SESI-HRMS measurements to avoid pollen-derived contamination in the headspace chamber. This final 24 h without pollen may have simplified the gut metabolic environment relative to natural hive conditions, where bees encounter a chemically complex and variable pollen diet; future work should test whether the same microbiota- and *Serratia*-associated volatile fingerprints remain detectable under more natural feeding conditions. The experimental setup in its current form lacks internal standards for normalization, as it does not involve any pressure-controlled flow-mixing chamber after the measurement tube (Fig. 4). The technique is also relatively low-throughput, especially when measuring one bee at a time (about 5 min per bee, including a preceding blank measurement). Its main application could therefore be identifying biomarkers to develop easy-to-use molecular sensors, more than real-time testing.

### Conclusions

Volatile metabolites provide a real-time, non-invasive window into host-microbe metabolism, and the honey bee is an ideal system to exploit this view. By combining single-bee SESI-HRMS with LC-HRMS, we show that gut microbiota markedly reshape the bee volatilome, enriching fatty-acid-associated signals and short-chain fermentation products that typify a healthy, colonized state. LC-HRMS and SESI-HRMS proved complementary, capturing largely non-overlapping chemical spaces and together revealing novel differentiators between microbiota-depleted and colonized bees. Extending this framework to infection, we demonstrate that *S. marcescens* imposes a distinct volatile fingerprint consistent with rerouting host pyruvate through the butanoate pathway; butane-2,3-diol and related pyruvate-derived metabolites emerge as candidates for a combinatorial volatile fingerprint of *Serratia* infection, although individual compound reliability remains limited. These results establish volatilomics as a practical tool for monitoring bee health and microbiome function and lay the groundwork for field-deployable chemical sensing to detect dysbiosis or pathogen pressure in managed pollinators.

## MATERIALS AND METHODS

### Generation of gnotobiotic honey bees

Late-stage pupae of *A. mellifera*, identified by their pigmented eyes and light gray cuticle, were aseptically removed from brood frames and transferred to sterile emergence boxes. Pupae were incubated at 35°C and 75% relative humidity for 3 days and supplied with sterile 50% (wt/vol) sucrose solution prepared in 1× PBS. Upon emergence, adult bees were fed irradiated pollen, which was removed 24 h before SESI-MS measurements to avoid pollen-derived contamination during analysis. Emerged bees were randomly assigned to one of four experimental groups: microbiota-depleted (MD), colonized with a SC, monocolonized with SM, or co-colonized with both the SCSM. All bacterial strains used in this study are listed in Supplemental material, with strain names, culture conditions, and genome accession information where available. After cultivation under the strain-specific conditions listed in Supplemental material, strains were washed and normalized to an optical density at 600 nm (OD_600_) of 1.0. Aliquots were frozen in 25% glycerol at −80°C until use. For colonization, each bee was individually hand-fed 5 μL of bacterial suspension diluted in 1× phosphate-buffered saline (PBS) supplemented with 50% (wt/vol) sucrose, using a micropipette to ensure delivery of the full standardized inoculum. SC group: Received a mix of 12 native gut strains at a combined final OD600 of 0.1. SM group: Received *S. marcescens* strain ESL0497 (submission ID: SAMN53263139) at OD_600_ = 0.1. SCSM group: Received the SC mix (OD_600_ = 0.1) supplemented with *S. marcescens* strain ESL0497 (OD_600_ = 0.1). MD group: Received a mock inoculum containing only the 1× PBS:50% sucrose vehicle. CFU counts were not remeasured after mixing inocula with sucrose. Bees were maintained under sterile conditions throughout the experiment and provided with autoclaved 50% (wt/vol) sucrose solution in water. Experiments were performed 5 days post-inoculation. For the *Serratia* experiment, *S. marcescens* recovery from SM and SCSM bees was checked qualitatively by selective plating at sampling. After SESI-MS measurements, bees were immediately stored at −80°C until further analysis. To minimize potential hive-specific effects, all experiments were biologically replicated using bees from four independent hives (*n* = 4). Where indicated, two technical replicates per treatment were also conducted using separate cages to account for cage-related variation.

### Real-time SESI-HRMS measurements of honey bee headspace

A set of air-tight transparent measurement tubes (2 cm diameter, 7 cm length, PMMA) was manufactured with Swagelok fittings and used as part of the in-house headspace sampling system (Fig. 4). Up to measurement, bees were housed in individual containers with free access to sterile irradiated pollen and sugar-water. Just before the experiment, the bees were first stunned using dry ice and then transferred to their measuring tube; 0.3 L × min^−1^ of humidified medical air (Pangas, Switzerland) was flushed through a carbon filter and into the bee tunnel, and subsequently introduced into the ion source. The humidifier, the bee tunnel, and all gas tubing in between were kept at 30°C ± 2°C. After the measurements, bees were anesthetized with carbon dioxide and decapitated. The guts were extracted for LC-HRMS analysis, and DNA extraction was performed within 30 min of headspace measurements.

### Real-time SESI-HRMS measurements of *S. marcescens* headspace

*S. marcescens* ESL0497 isolates were grown on Brain Heart Infusion supplemented with sheep blood (BHI-S) plates, and single colonies were then chosen as replicates for growth in liquid Sf-900 insect cell culture media. The cultures were grown either under aerobic or fully anaerobic conditions, with three replicates per condition. The replicates were sampled during exponential growth (OD_600_ = 0.3) and transferred into sealed round-bottom flasks. To sample the headspace, 0.3 L × min^−1^ of humidified carrier gas (9:1 mixture of nitrogen to carbon dioxide, Pangas, Switzerland) was flushed through the round-bottom flask containing 3 mL of the bacterial culture and introduced into the SESI source. The humidifier, the headspace sampler, and all gas tubing in between were kept at 30°C ± 2°C.

### Data acquisition by SESI-HRMS

Data were acquired using a commercial SESI source (Fossil Ion Tech, Spain) coupled to a Q Exactive Plus Orbitrap high-resolution mass spectrometer (Thermo Fisher Scientific, Germany). During measurements, a stable vacuum pressure of ca. 8 × 10^−11^ mbar was maintained. The SESI sampling line and the ionization chamber were heated at 130°C and 90°C, respectively. For each measurement, both positive and negative ionization modes were recorded separately. The electrospray was generated using a 0.1% aqueous (LC-MS grade water, Thermo Fisher Scientific) formic acid solution (99.5% formic acid, VWR Chemicals, Switzerland), passed through an electrospray capillary (outer diameter = 365 µm, inner diameter = 20 µm) under a positive pressure of 800 mbar. The sheath gas was kept at 15 a.u., and the auxiliary gas at 2 a.u. The electrospray voltage was set to ± 3.5 kV, depending on the polarity mode. The MS inlet capillary was heated to 250°C. Automatic gain control in the C-trap was set to 1e6, and the maximum injection time to 500 ms. The mass resolution was kept at 140,000 for both full-MS and MS/MS scans, and the S-lens RF level was set to 50. For each polarity mode, MS1 spectra were recorded in profile mode, followed by 10 consecutive data-dependent MS2 scans in centroid mode.

In total, 32 individual honey bees were analyzed for the MD/SC comparison (*n* = 16 per group: 4 hives × 2 cages × 1 bee per cage and condition). For the SM/SCSM experiments, four biological replicates were measured per group, each consisting of three individual bees, wherein a single bee was measured at a time (*n* = 12 per group). Once proper airflow, airtightness, and heating were ensured, the measurement was started for a total of 4 min: 60 s for a full MS1 acquisition in positive ion mode, another 60 s for the negative ion mode, 60 s for ddMS (2) measurements in positive ion mode, and 60 s in the negative ion mode. Additionally, blank measurements of the background ions present in humidified medical air passed through the measurement tube were taken before every replicate.

### Metabolite extraction for LC-HRMS

After excision, the honey bee gut samples were transferred into tubes containing zirconia beads (0.1 mm diameter zirconia/silica beads, Carl Roth, Switzerland). The wet weight of each sample was taken, and 10× (vol/wt) Optima LC-MS grade water (Fisher Scientific, Switzerland) was added. The samples were homogenized using a Retsch MM 400 homogenizer at 30 Hz for 30 s. The samples were then split into two fractions: one for metabolomics by LC-HRMS and one for DNA extraction. The metabolomics aliquots of 100 µL were further diluted 1:1 with Optima water. Following a previously established protocol (23, 23), the samples were incubated at 80°C and 1,400 rpm for 3 min, additionally vortexing the samples after each minute. This procedure was then followed by 5 min of centrifugation at 20,000 × *g* at 4°C, after which 150 µL of the supernatant was collected and centrifuged under the same conditions for another 30 min. Finally, the samples were again 10× diluted in LC-MS grade water and stored at −80°C.

### 16S rRNA gene sequencing of the gut bacterial loads and quantification of bacterial loads by quantitative PCR (QPCR)

The homogenized samples for DNA extraction were processed using phenol-chloroform extraction and sodium acetate precipitation as described previously (23). The DNA quality was assessed using the 4200 TapeStation System (Agilent Technologies, Switzerland). Full-length amplicon sequencing of the 16S rRNA gene was performed using the PacBio Revio platform (Novogene, Germany). Universal primers 27F (5′-AGRGTTYGATYMTGGCTCAG-3′) and 1492R (5′-RGYTACCTTGTTACGACTT-3′) targeting the full-length 16S rRNA gene were used for amplification. The PacBio BAM file was split according to the barcode and quality-filtered using default QIIME2 (v2022.03) parameters. Amplicon sequence variants (ASVs) were generated and taxonomically assigned using the Greengenes reference database. Prediction of metagenomic functions was performed using the PICRUSt2 algorithm (bundled with QIIME2 2022.03), which infers functional gene content from 16S rRNA gene-based taxonomic profiles using Greengenes-mapped reference genomes. We note that PICRUSt2 predictions are most reliable for well-characterized taxa and may underestimate the functional diversity of poorly represented lineages.

qPCR was performed on a QuantStudio 5 system (Applied Biosystems) in 96-well plates, using 10 μL reactions with 0.2 μM primers, 1× SYBR Select Master Mix, and 1 μL DNA template. Thermal cycling included 50°C for 2 min, 95°C for 2 min, followed by 40 cycles of 95°C for 15 s and 60°C for 1 min. Each sample was run in triplicate. Total bacterial load was assessed using universal 16S primers 356F (AGGATTAGATACCCTGGTAGTCC) and 772R (YCGTACTCCCCAGGCGG), and host DNA was quantified with actin primers 0113/0114 for normalization. Samples with actin Cq > 28 or no actin amplification were excluded. Copy numbers were calculated from standard curves (universal 16S assay: *E* = 1.945, *R*^2^ = 0.999, intercept = 36.1153038; actin assay: *E* = 2.022, *R*^2^ = 1.000, intercept = 35.094212), multiplied by the total gut homogenate volume (300 μL), and normalized as (16S copies/actin copies) × median(actin copies).

### Data acquisition by LC-HRMS/MS

Samples were injected into a Vanquish Flex (Thermo Fisher Scientific, Germany) liquid chromatograph coupled to a heated electrospray ion source (H-ESI) of an Orbitrap Exploris 240 mass spectrometer (Thermo Fisher Scientific, Germany). Hydrophilic interaction chromatography was performed on a Waters Acquity BEH HILIC Column (1.7 µm, 2.1 mm × 100 mm). The mobile phase was a gradient mixture of ammonium formate (2 mM, aqueous), formic acid (0.1% [vol/vol], pH 3), and acetonitrile. The HILIC gradient was as follows (flow rate: 0.5 mL/min): 0–1 min 98% B, 1–5 min 98%–80% B, 5–8.5 min 80%–40% B, 8.5–10 min 40% B, 10–10.1 min 40%–98% B, 10.1–15 min 98% B (re-equilibration), where mobile phase A was 10 mM ammonium formate + 0.1% formic acid in water and B was acetonitrile. Absorbance data for each run were collected at 254 nm and 280 nm. The mass spectrometer was operated in both the positive and negative ionization modes, each recorded in a separate run. In both modes, the sheath gas was set to 30 a.u., the auxiliary nebulizer gas to 10 a.u. at 400°C, and the ion transfer tube was heated to 350°C. The S-lens RF voltage was kept at 0. In positive mode, the capillary voltage was set to 3.4 kV, and in negative mode, to 2.5 kV. Data acquisition was performed with full-MS (50–750 *m/z*) scans recorded in profile mode, followed by a series of 20 consecutive data-dependent MS/MS (ddMS) (2) scans recorded in centroid mode, with an intensity threshold and a 60 s dynamic exclusion window filter. The ddMS (2) scans were acquired with stepped normalized collision energies of 5, 30, 70, 150, and 200. In both full-MS and MS/MS scan modes, the mass spectrometer resolution was set to 120,000 with a standard (1e6) AGC target and automatic injection time adjustment.

### Data processing

Raw MS data files in the proprietary Thermo Fisher Scientific RAW file format were first converted into the open mzML format using MSConvert (ProteoWizard package) (2). Raw LC chromatograms were directly exported from the Chromeleon Chromatography Data System in the TXT file format. The mzML and TXT files were subsequently analyzed using LCMSpector, a standalone user-interface application for viewing and analyzing LC-MS data (3). The processed CSV files, containing all the targeted primary ions, their absorbance values and MS intensities, as well as concentrations extrapolated from calibration curves of standard samples, were then exported and further analyzed with R. All data visualization and post-processing scripts are provided as an organized R analysis repository, available at https://doi.org/10.5281/zenodo.19608467.

Data-dependent acquisition tandem mass spectra (2) were processed and annotated with SIRIUS (54). Detailed information containing the putative assignments of fragment ions can be found in the Supplemental material.

### Statistical analysis

Differential abundance between groups was assessed using Welch’s *t*-test. For targeted analyses with a small number of comparisons, *P*-values were corrected using the Holm-Bonferroni method; for untargeted analyses involving thousands of features, the Benjamini-Hochberg false discovery rate correction was applied. Features were considered significant at adjusted *P* <0.05 and absolute fold change >2. Multivariate analysis was performed using PLS-DA (mdatools R package) with two components and scaling enabled. Pathway enrichment for the three enrichment panels (Fig. 1D through 3C) was performed using the Mummichog module of MetaboAnalyst 6.0 (55). For each comparison, a tab-delimited peak list (*m/z*, *P*-value, *t*-statistic, and ion mode) was generated by per-feature Welch’s *t*-tests on log2-transformed intensities (paired-blank-subtracted where blanks were available: Fig. 1D and the bee-headspace component of 3C). Mummichog was run at Orbitrap 5 ppm mass accuracy in mixed ion mode with a per-feature *P* < 0.05 cutoff against an organism-appropriate KEGG library: *A. mellifera* (“ame” for the bee-derived data sets (Fig. 1D and 2G and the green bars of Fig. 3C) and *S. marcescens* (“smar”) for the *Serratia* culture data set (the magenta bars of Fig. 3C). Pathways are ranked and colored by the Mummichog gamma *P*-value, which accounts for uncertainty in *m*/*z*-to-compound matching; the top-ranked pathways are shown per panel. To account for potential confounding from experimental design, linear mixed-effects models (lme4 R package) were fitted with treatment group as a fixed effect and hive of origin as a random effect.

## Data Availability

The original data and analysis code used in this publication are publicly available in a curated Zenodo archive at https://doi.org/10.5281/zenodo.19608467.

## SUPPLEMENTAL MATERIAL

File S1 mbio.01419-26-s0001.xlsx Reference list of literature metabolites discovered differentiated in SC and MD bees.

File S2 mbio.01419-26-s0002.xlsx Bacterial strains comprising the Synthetic Community with public genome accession numbers.

File S3 mbio.01419-26-s0003.csv SESI-HRMS feature annotations for the MD/SC experiment.

File S4 mbio.01419-26-s0004.csv *m*/*z* features comprising the *Serratia* headspace signature.

File S5 mbio.01419-26-s0005.csv SESI-HRMS feature annotations for the SC/SM experiment.

Supplemental material mbio.01419-26-s0006.pdf Supplemental figures and tables.

ASM does not own the copyrights to Supplemental Material that may be linked to, or accessed through, an article. The authors have granted ASM a non-exclusive, world-wide license to publish the Supplemental Material files. Please contact the corresponding author directly for reuse.
