# Supplementary material for: Real-time volatilomics reveals microbiota and pathogen fingerprints in the honey bee
Source: mBio. 2026 Sep 9;17(10):e01419-26. doi: 10.1128/mbio.01419-26 (PMC13712819; doi:10.1128/mbio.01419-26)
Supplement: Supplementary file 1 [file mbio.01419-26-s0006.pdf]

Supporting Information for the manuscript

“Real-time volatilomics reveals microbiota and pathogen fingerprints in the honey bee”

Table of contents

Table S1. Composition of the defined bacterial community present in SC bees.

Table S2. Putative annotations of differentiating SESI-HRMS features in the MD-vs-SC bee headspace comparison.

Fig. S1. Average abundances of top 20 most abundant bacterial strains found in the guts of MD and SC bees.

Fig. S2. Relative taxonomic distribution in gut and carcass samples of SC and MD bees.

Fig. S3. Volcano plot of species abundance in SC and MD bee guts.

Functional analysis

Fig. S4. Metabolic pathways corresponding to species found within SC and MD bee guts.

Fig. S5. Volcano plot of putative metabolic pathway upregulation.

Headspace measurements of liquid cultures of S. marcescens by SESI-HRMS

Fig. S6. PCA plot of liquid cultures of S. marcescens grown in aerobic and anaerobic conditions.

Fig. S7. Heatmap of m/z intensities in S. marcescens cultures.

Fig. S8. Key metabolites in the butanoate pathway and TCA cycle in medium and S. marcescens cultures.

Serratia-colonized bees measured by SESI-HRMS

Fig. S9. Heatmap of bee headspace samples from SM, SCSM, and SC groups.

Fig. S10. Multivariate analysis of the Serratia-infected bee headspace profiles (SC, and SM) limited to the signature 221 features, measured by SESI-HRMS.

Fig. S11. Multivariate analysis of the Serratia-infected bee headspace profiles (SC, SCSM, SM) measured by SESI-HRMS.

Fig. S12-S23. Serratia signature-feature boxplots.

Fig. S24. PCA of the 221-feature Serratia signature in SC, SCSM, and SM bees.

Fig. S25. qPCR bacterial loads across MD and SC gut samples.

Synthetic community (SC) composition

Table S1. Composition of the defined bacterial community present in SC bees.

Bacterial species	Core member identifier	Strain designation	Growth and incubation conditions	Public genome accession(s)
Snodgrassella alvi	Snod-1	wkB2 / ESL0145	BHI; micro-aerophilic, 35oC; 24-48 h	GCA_000600005.1; CP007446
Gilliamella apis	Gilli-2	ESL0169	BHI; micro-aerophilic, 35oC; 24-48 h	GCA_003202875.1; QGLN00000000
Gilliamella apicola	Gilli-1	ESL0178	BHI; micro-aerophilic, 35oC; 24-48 h	GCA_003202815.1; QGLQ00000000
Frischella perrara	Frischella	ESL0034	BHI; anaerobic, 35oC; 24-48 h	IMG: Ga0077910; no public INSDC genome accession found
Bifidobacterium indicum / B. coryneforme	Bifido-2	ESL0197	BHI; anaerobic, 35oC; 24-48 h	GCA_003202665.1; QGLI00000000
Bifidobacterium polysaccharolyticum	Bifido-1.4.1	ESL0198	BHI; anaerobic, 35oC; 24-48 h	GCA_003202715.1; QGLJ00000000
Lactobacillus apis	Firm5-1	ESL0185	BHI; anaerobic, 35oC; 24-48 h	GCA_003150935.1; CP029476
Lactobacillus helsingborgensis	Firm5-2	ESL0183	BHI; anaerobic, 35oC; 24-48 h	GCA_003173695.1; CP029544, CP029545
Lactobacillus melliventris	Firm5-3	ESL0184	BHI; anaerobic, 35oC; 24-48 h	GCA_003202825.1; QGLG00000000
Lactobacillus kullabergensis	Firm5-4	ESL0186	BHI; anaerobic, 35oC; 24-48 h	GCA_003151025.1; CP029477
Bombilactobacillus mellis	Firm4-1	ESL0294	BHI + 10% cfMRS; anaerobic, 35oC; 24-48 h	GCA_013346925.1; JAAEEA000000000
Commensalibacter melissae	Alpha2.1	ESL0284	BHI; anaerobic, 35oC; 24-48 h	GCA_003202795.1; QGLT00000000

Genome accessions are listed where a public genome record could be identified. For Frischella perrara ESL0034, only the JGI IMG genome ID Ga0077910 was found in public strain-genome listings; no public INSDC/GenBank assembly accession was identified.

Table S2. Putative annotations of differentiating SESI-HRMS features in the MD-vs-SC bee headspace comparison.

m/z	mode	Neutral mass	Formula	Mass error (ppm)	DBE	annotation	log2fc	p
-283.2642	neg	284.27	C18H36O2	1.7	1	Stearate	15.5	1.10E-07
133.0494	pos	132.04	C5H8O4	5.2	2	Glutaric acid	-12.2	4.20E-06
97.0283	pos	96.02	C5H4O2	6.8	4	Furfural	-13.3	2.50E-05
-255.2329	neg	256.24	C16H32O2	1.9	1	Palmitate	13.3	2.70E-05
-256.2363	neg	257.24	13C-C16H31O2	0.4	NA	[13C]Palmitate	9.6	7.60E-04
-256.2363	neg	257.24	13C-C16H31O2	0.4	NA	[13C]Palmitate	9.6	7.60E-04
-151.0233	neg	152.03	C4H8O6	6.3	1	Tartaric acid	-8.9	1.80E-04
123.0551	pos	122.05	C6H6N2O	6	5	Nicotinamide	-8.1	3.40E-04
-87.0451	neg	88.05	C4H8O2	5.6	1	Butyrate	4.6	4.80E-02
-59.0137	neg	60.02	C2H4O2	6.6	1	Acetate	5.1	7.30E-02
-73.0295	neg	74.04	C3H6O2	7.4	1	Propionate	3.8	7.70E-02
85.0283	pos	84.02	C4H4O2	7.8	3	2(3H)-Furanone	-14	2.30E-05
101.0232	pos	100.02	C4H4O3	6.7	3	Succinic anhydride	-11.9	5.60E-05
108.0520	pos	107.04	NA	NA	NA	NA	-8.8	6.30E-05
-75.0005	neg	76.01	C2H4O3	8	1	Glycolic acid	-10.3	1.40E-04
117.0545	pos	116.05	C5H8O3	5.8	2	5-Hydroxypentanoate	-11.1	2.00E-04
237.0728	pos	236.07	C7H12N2O7	2.2	3	NA	9.8	4.60E-04
271.2987	pos	270.29	C18H38O	5.1	0	Octadecanol	-9	5.10E-04
227.0885	pos	226.08	C6H14N2O7	2.5	1	NA	10.5	5.30E-04
275.0795	pos	274.07	C12H10N4O4	5.4	10	NA	9.3	6.10E-04
-92.0070	neg	93.01	NA	NA	NA	NA	6.7	7.40E-04

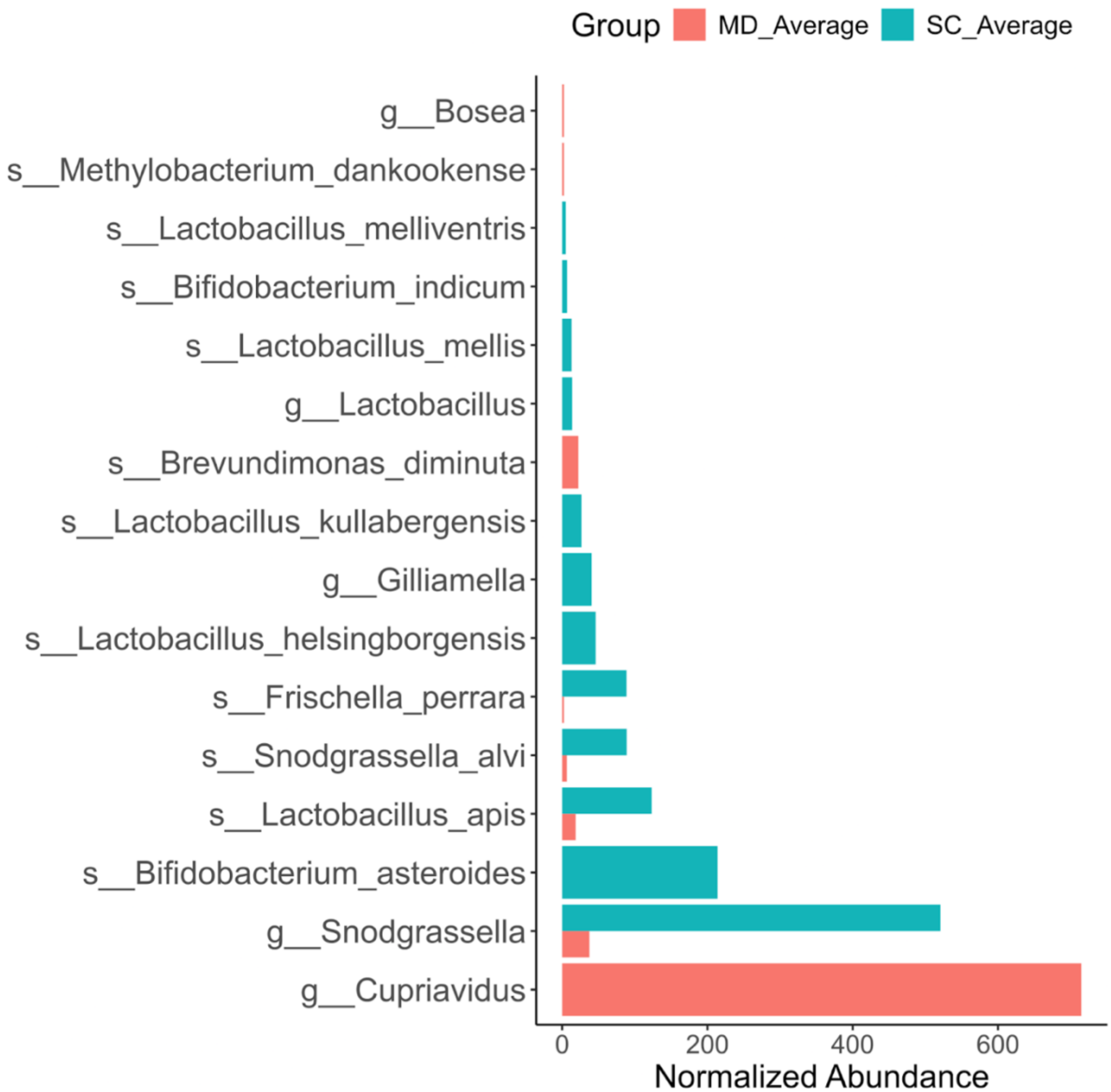

Fig. S1. Average abundances of top 20 most abundant bacterial strains found in the guts of MD and SC bees by 16S PacBio full-length sequencing. Abundances are normalized to CV values obtained for every sample by qPCR.

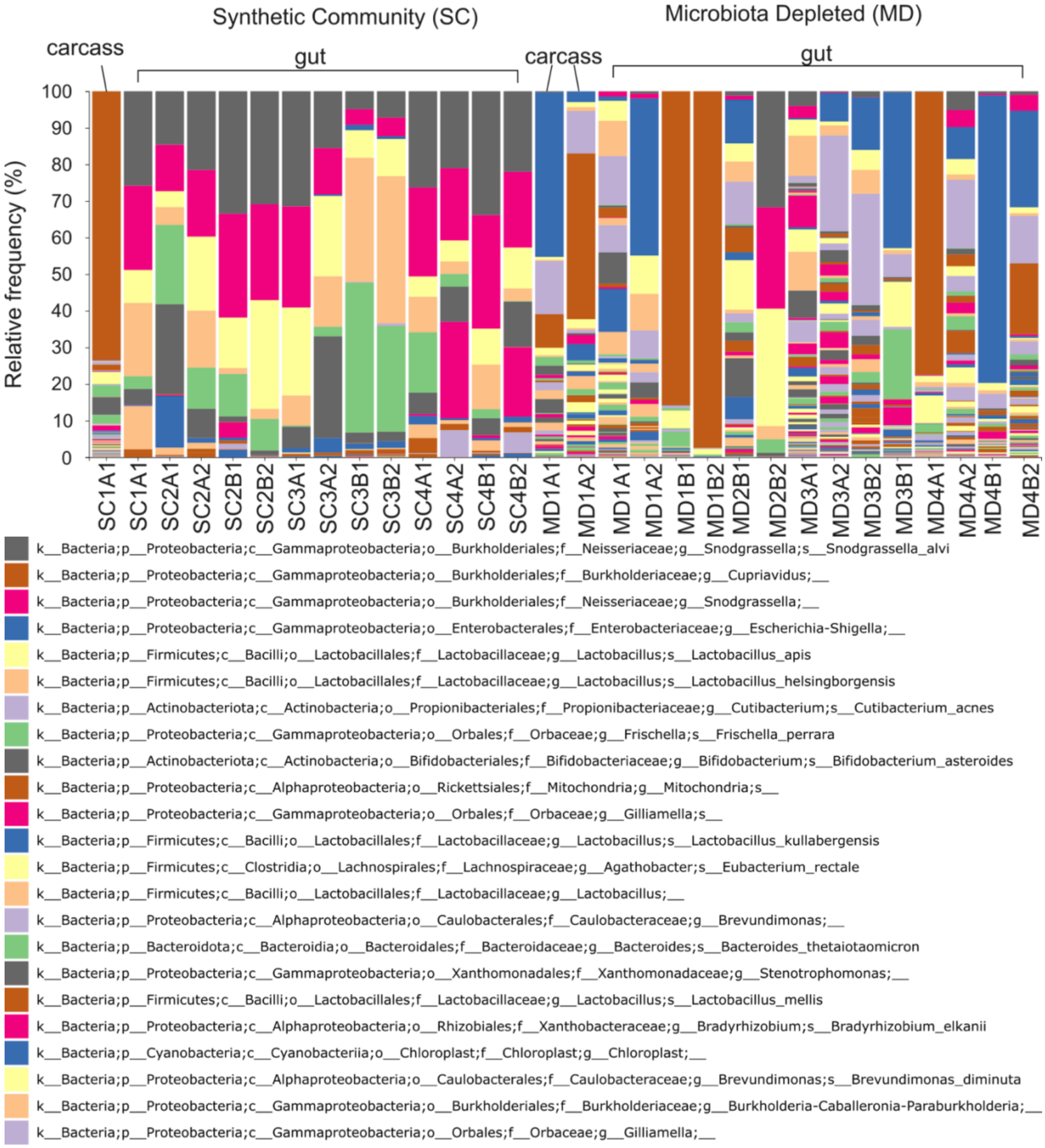

Fig. S2. Relative taxonomic distribution in the gut and carcass samples of SC and MD bees on the level of the species level by PacBio full-length 16S sequencing.

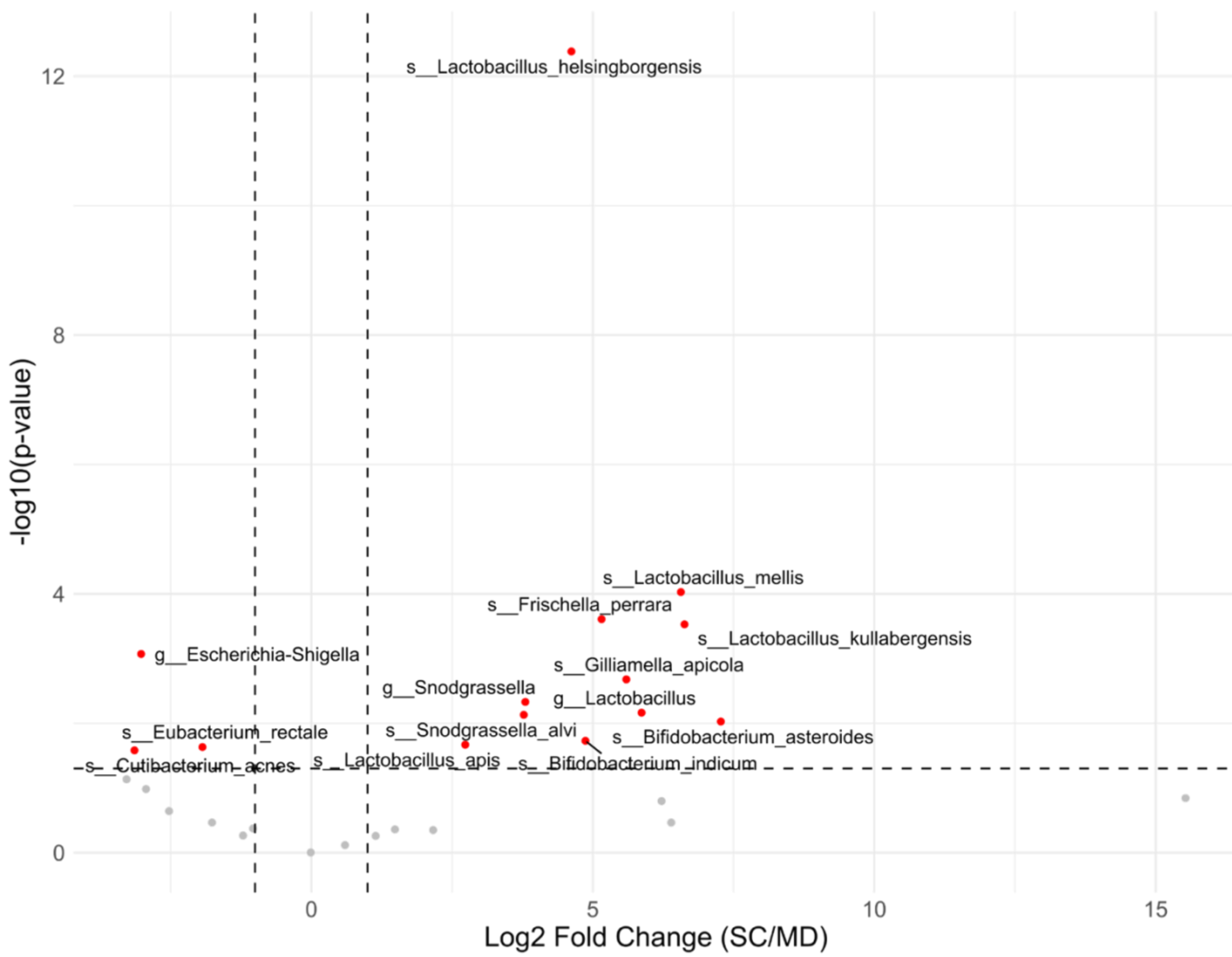

Fig. S3. Volcano plot of species abundance in SC and MD bee guts, normalized to qPCR CV values for each sample.

Functional analysis

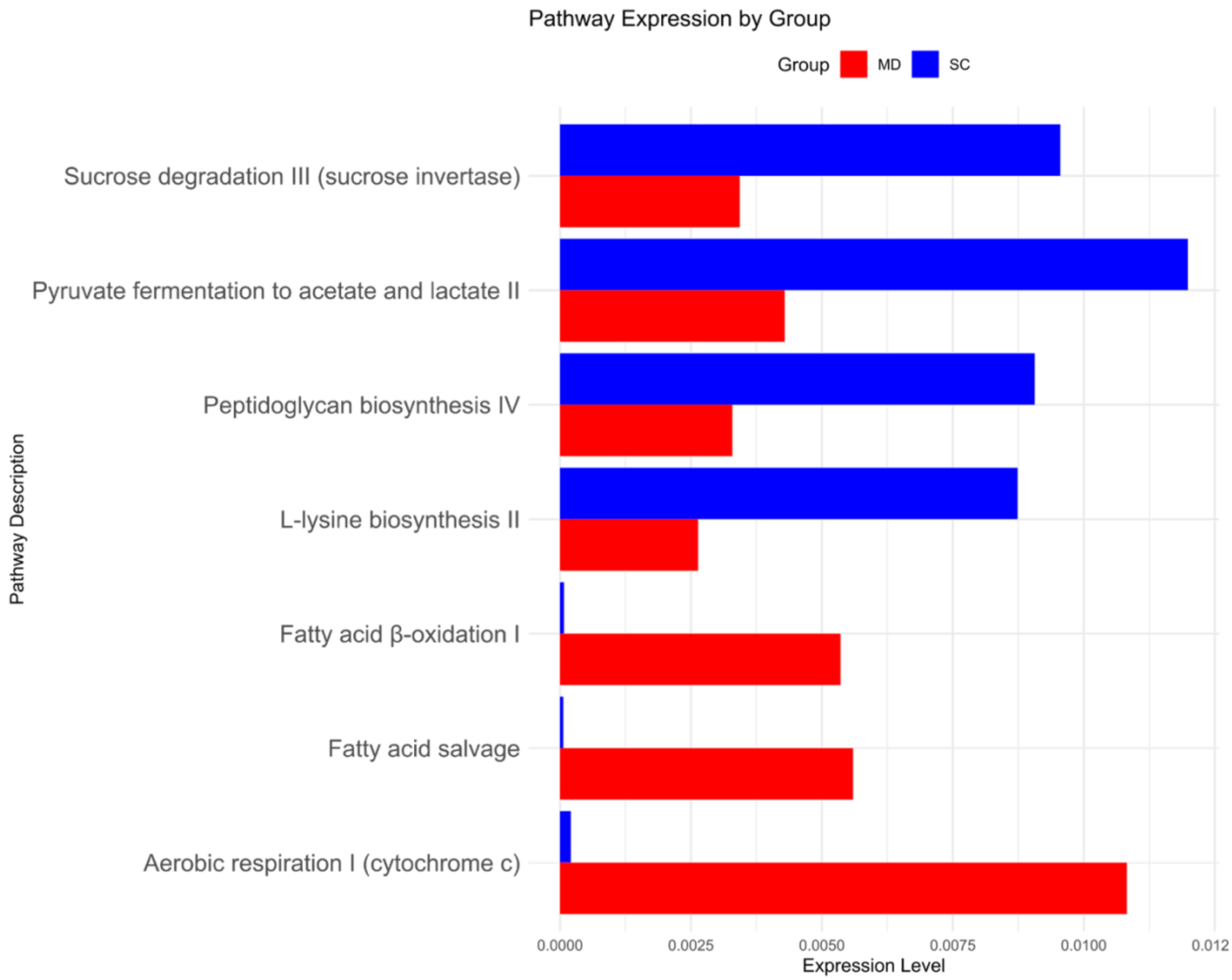

Fig. S4. Metabolic pathways corresponding to the species found within SC and MD bee guts by full-length 16S PacBio sequencing as a function of expression level.

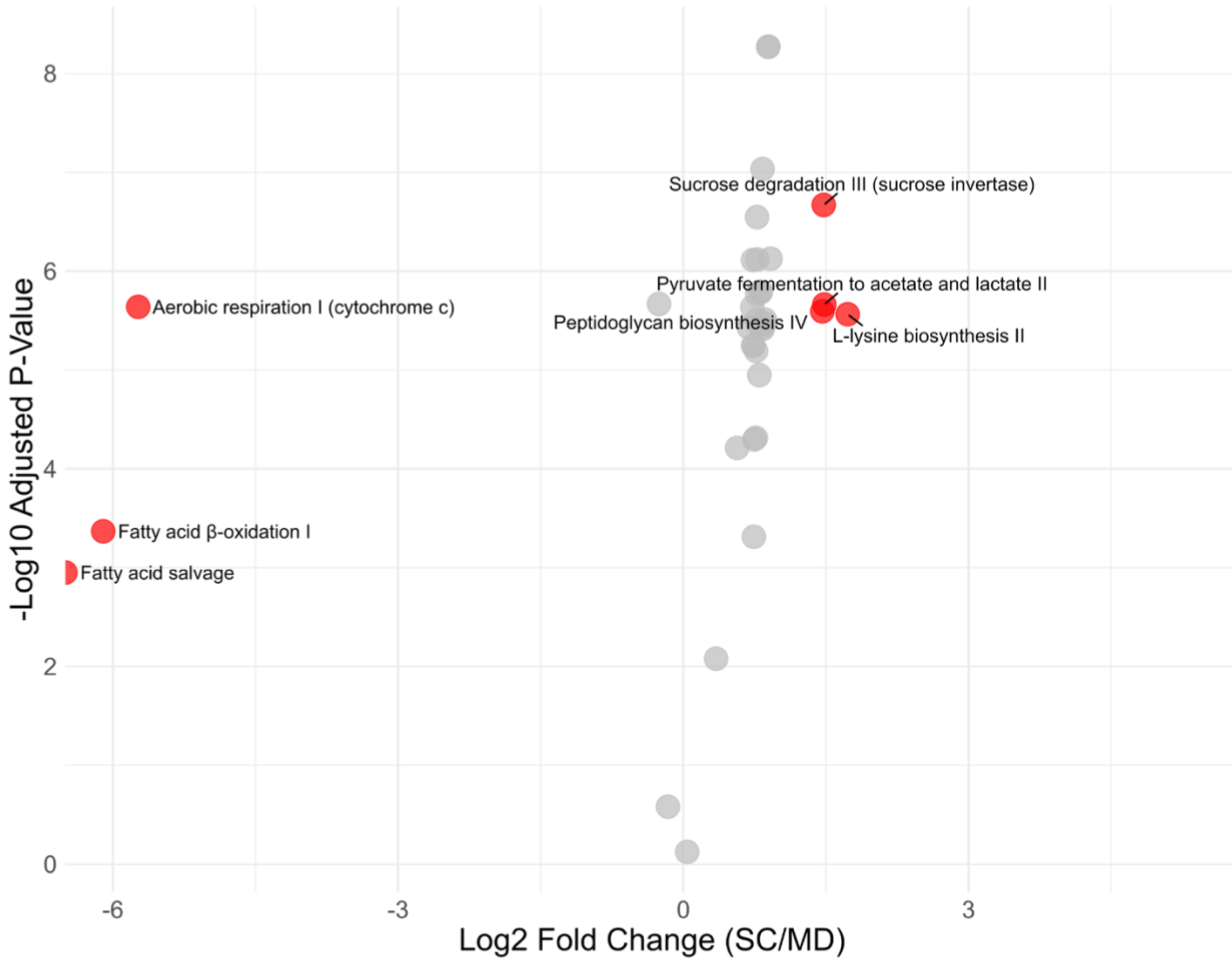

Fig. S5. Volcano plot of putative metabolic pathway upregulation as per expression levels and fold change of species abundance in SC and MD bee guts by full-length 16S PacBio sequencing.

Headspace measurements of liquid cultures of S. marcescens by SESI-HRMS

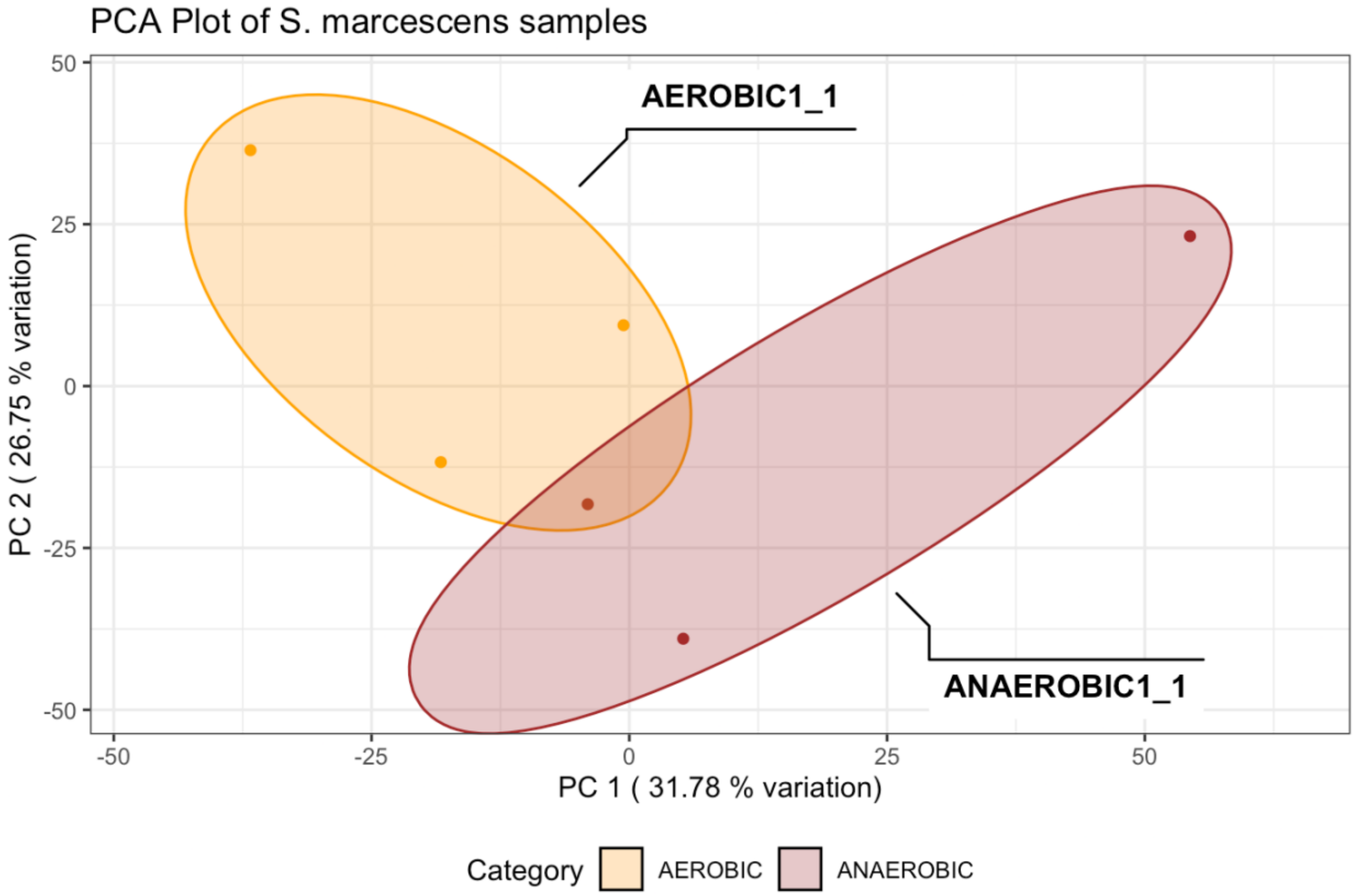

Fig. S6. PCA plot showing the distribution of headspace samples of liquid cultures of S. marcescens grown in aerobic and fully anaerobic conditions (3 biological replicates each), measured by SESI-HRMS.

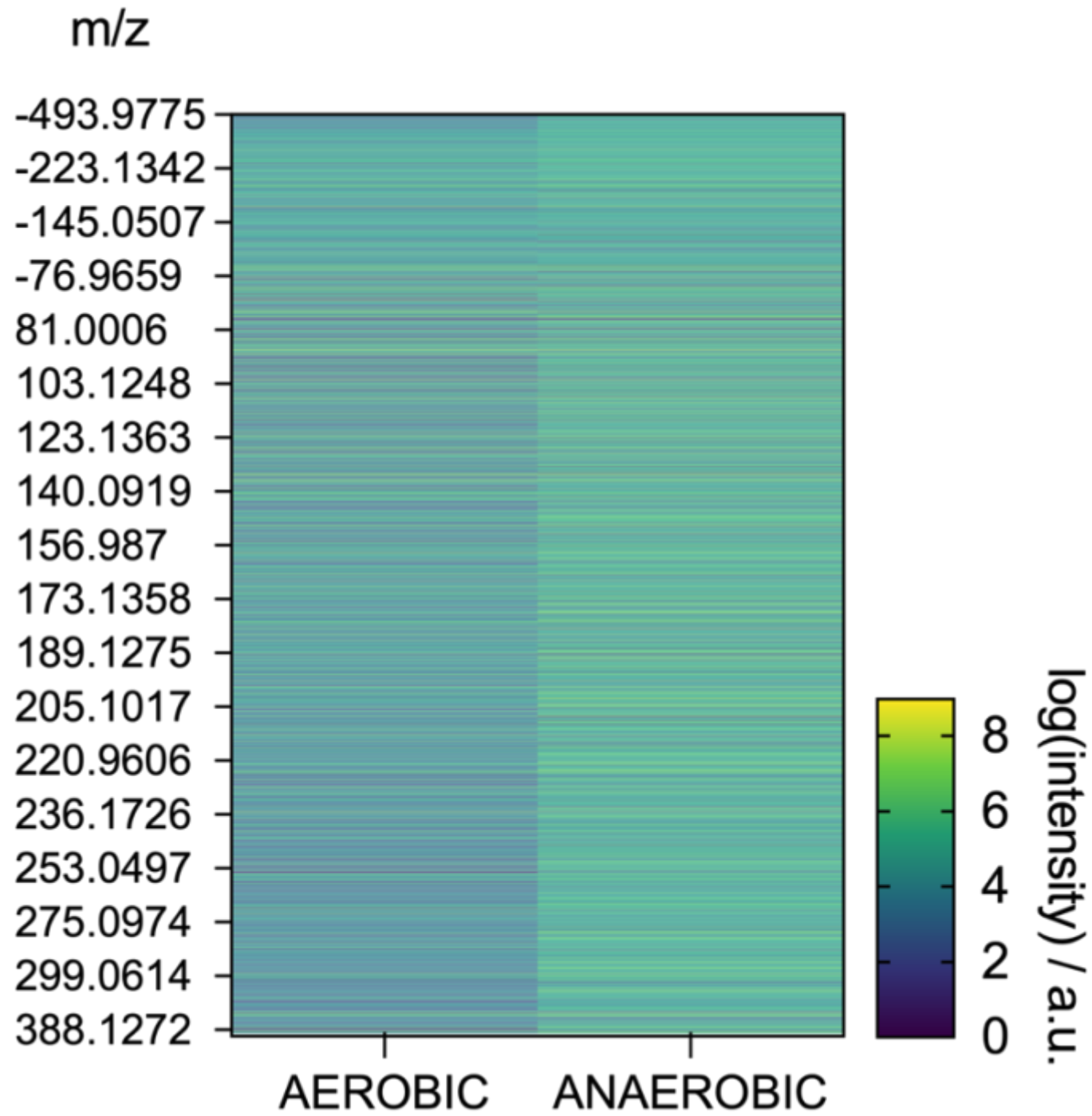

Fig. S7. Heatmap showing the logarithmic intensities of m/z values in aerobically and anaerobically grown S. marcescens cultures.

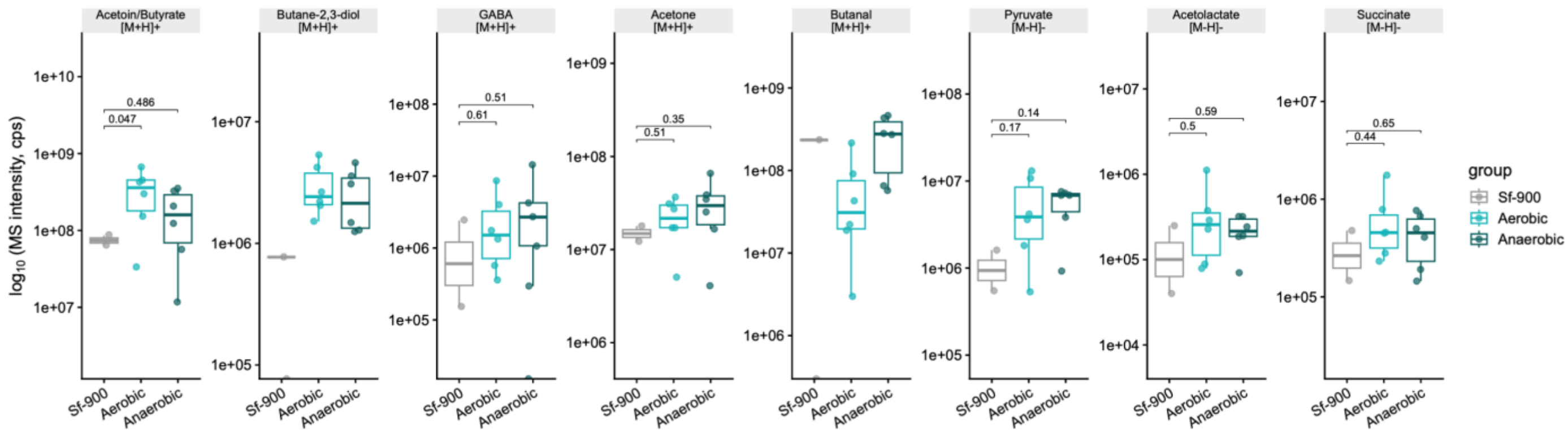

Fig. S8. Boxplots showing absolute MS intensities in counts per second for targeted S. marcescens liquid-culture compounds detected in positive and negative ionization modes by SESI-HRMS. Aerobic and anaerobic culture profiles are shown together with the sterile Sf-900 medium control.

Serratia-colonized bees measured by SESI-HRMS

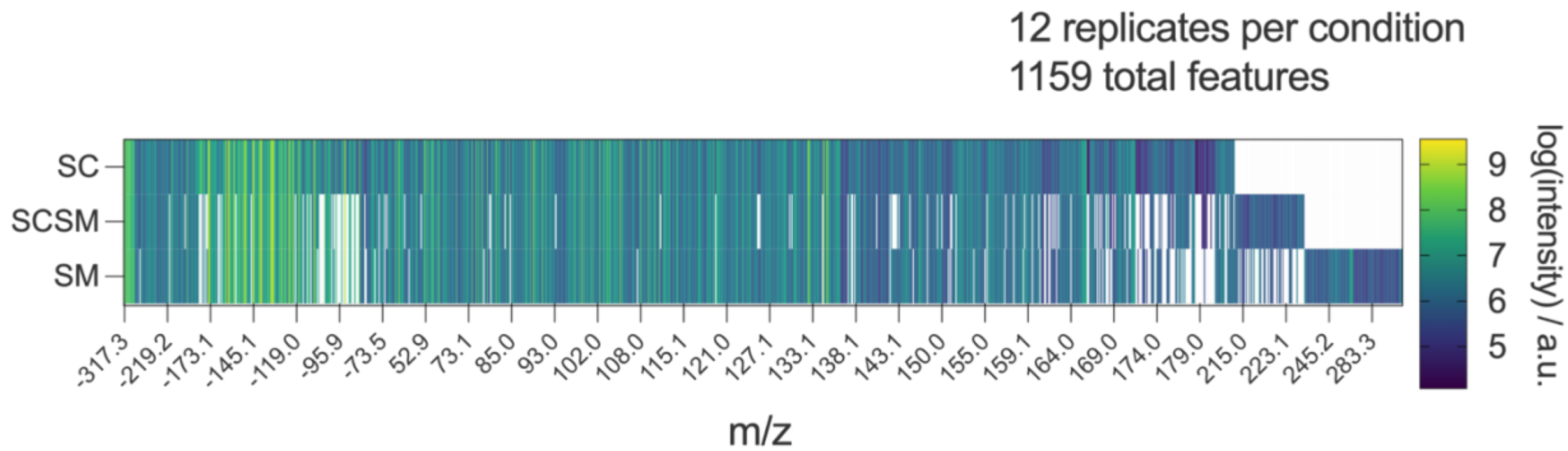

Fig. S9. Heatmap showing the logarithmic intensities of m/z values in headspace samples of bees colonized only with S. marcescens (SM), colonized with S. marcescens and a defined synthetic gut community (SCSM), and only colonized with a defined synthetic gut community (SC).

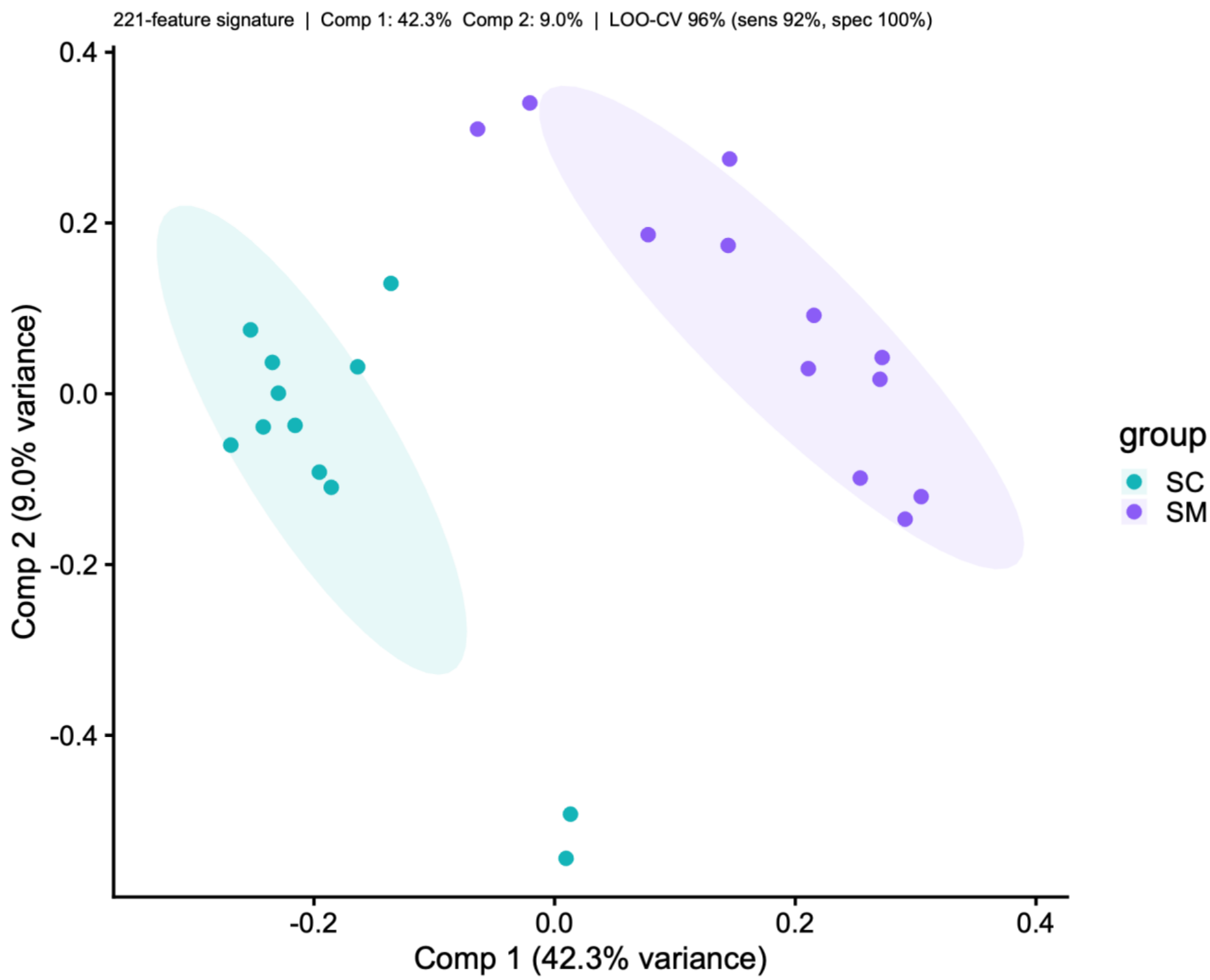

Fig. S10. Multivariate analysis of the Serratia-infected bee headspace profiles (SC, and SM) limited to the signature 221 features, measured by SESI-HRMS.

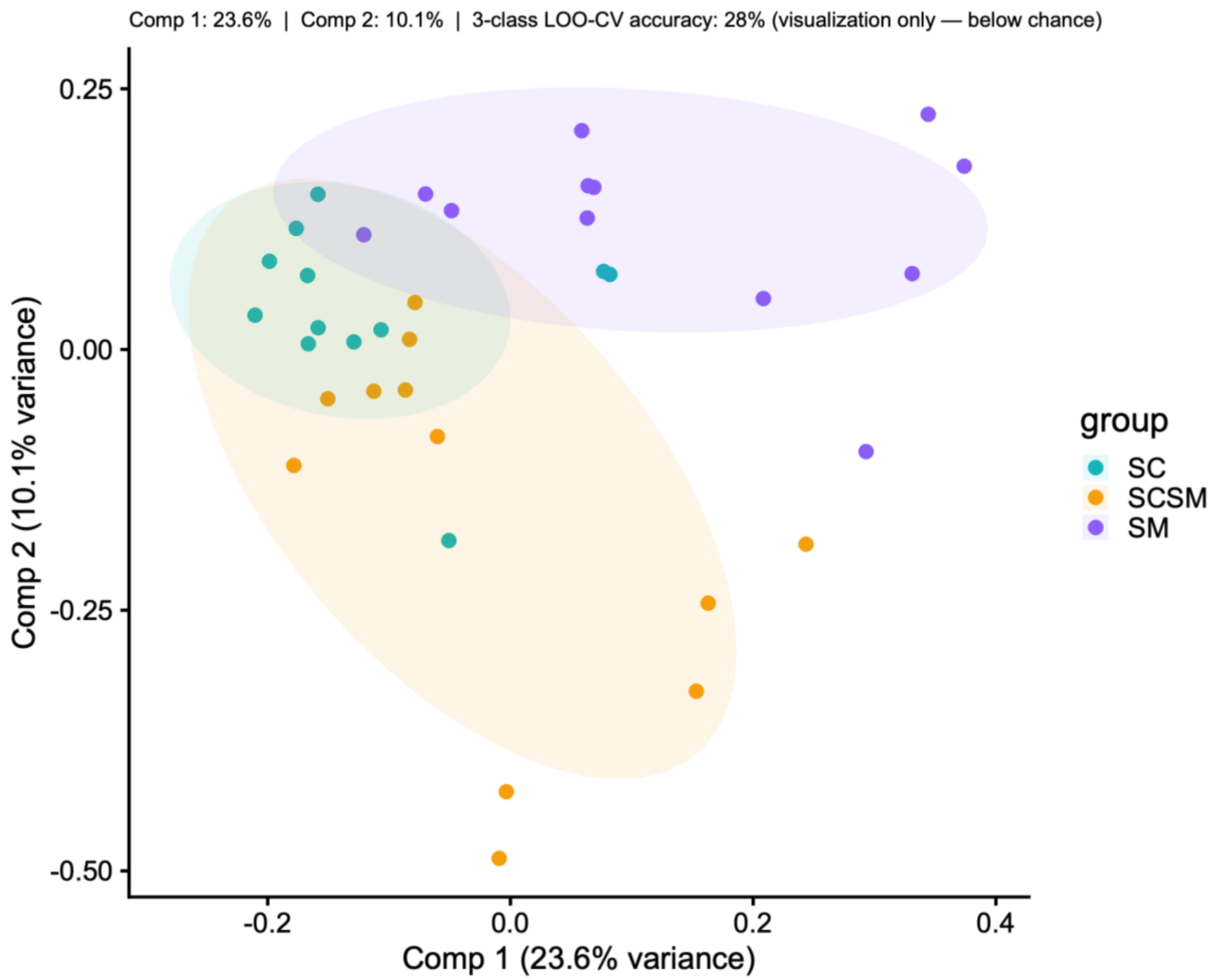

Fig. S11. Multivariate analysis of the Serratia-infected bee headspace profiles (SC, SCSM, SM) measured by SESI-HRMS.

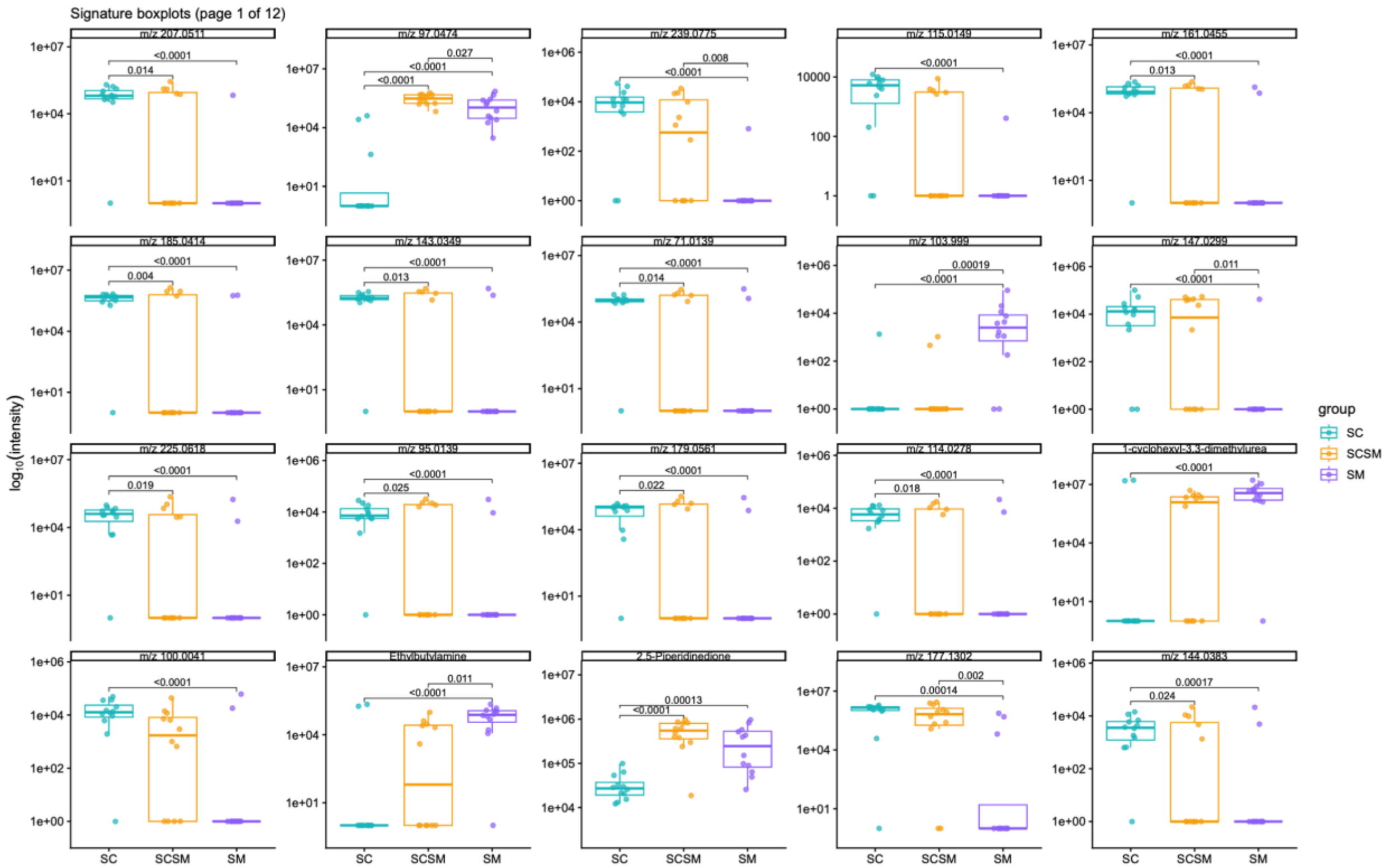

Fig. S12. Boxplots showing the absolute MS intensities in counts per second of the 221 features composing the Serratia signature from the PLS-DA algorithm (part 1 out of 12).

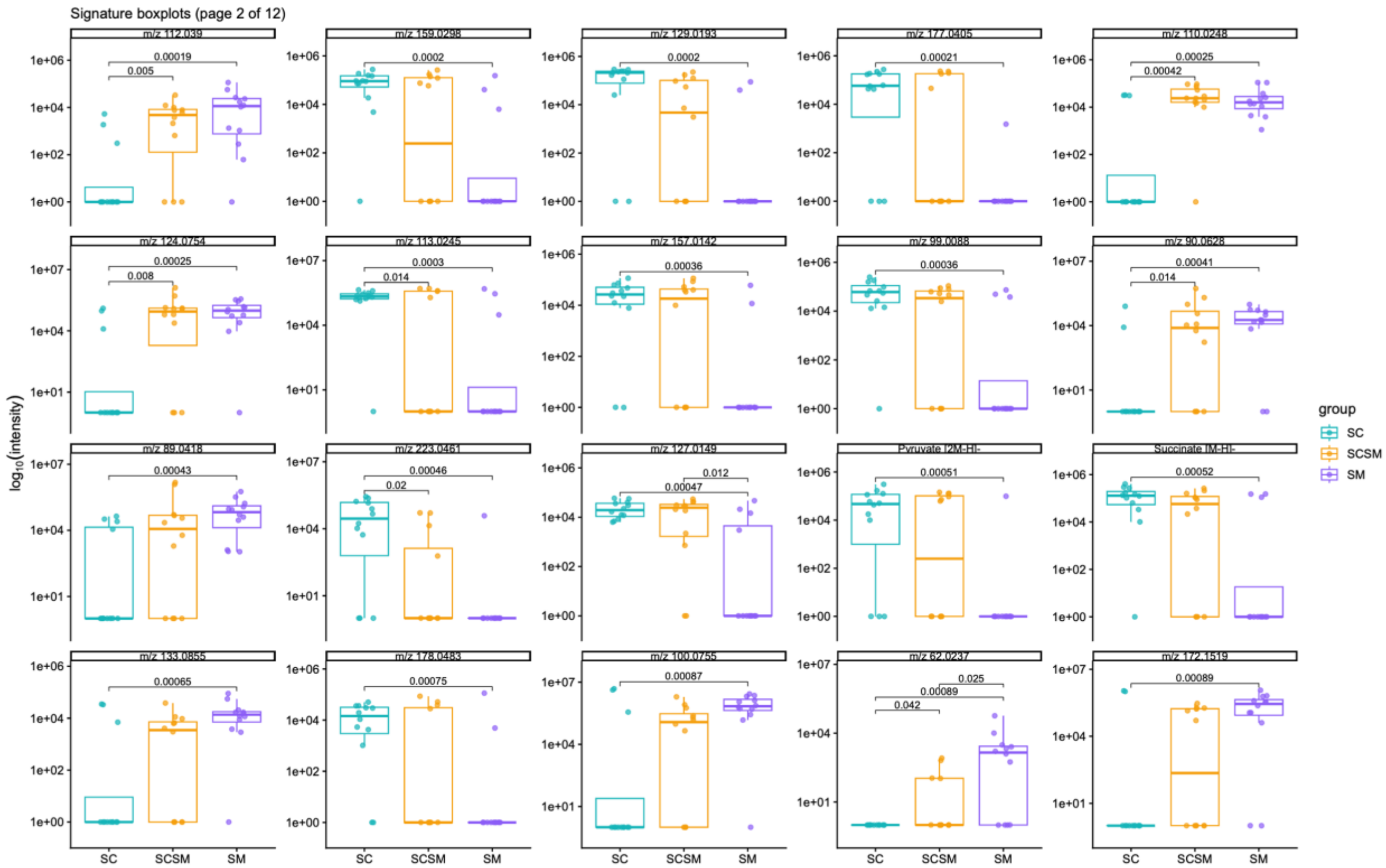

Fig. S13. Boxplots showing the absolute MS intensities in counts per second of the 221 features composing the Serratia signature from the PLS-DA algorithm (part 2 out of 12).

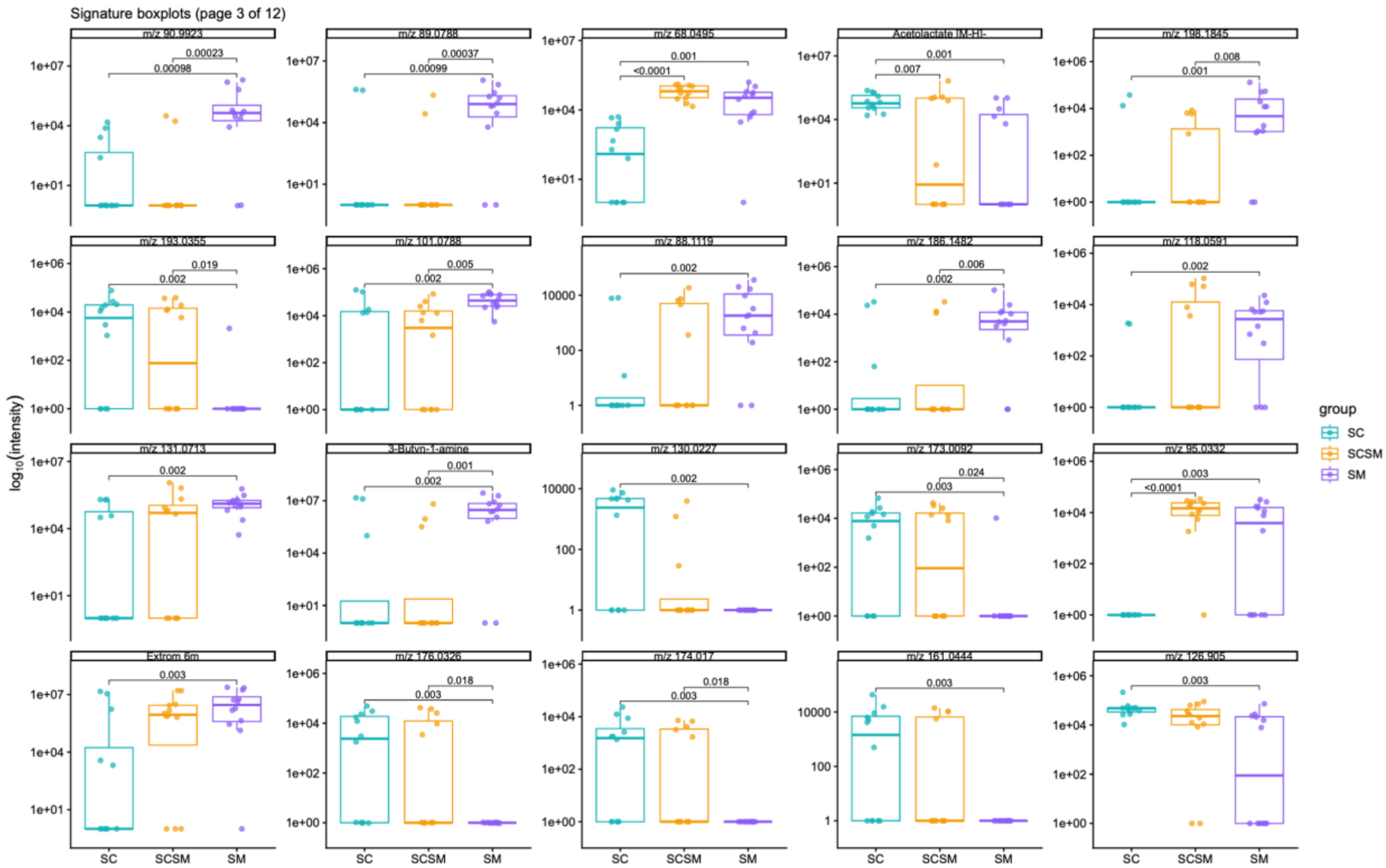

Fig. S14. Boxplots showing the absolute MS intensities in counts per second of the 221 features composing the Serratia signature from the PLS-DA algorithm (part 3 out of 12).

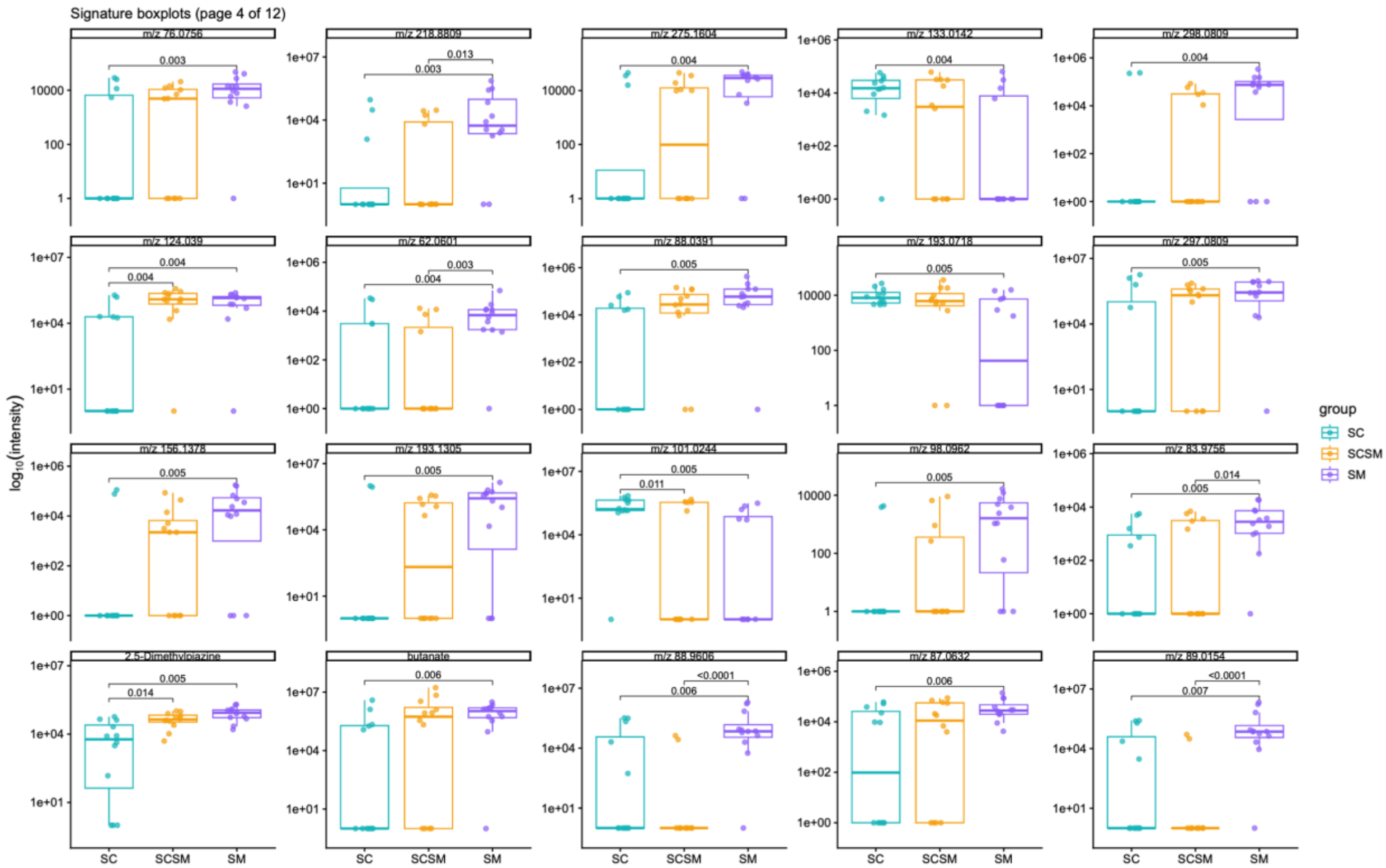

Fig. S15. Boxplots showing the absolute MS intensities in counts per second of the 221 features composing the Serratia signature from the PLS-DA algorithm (part 4 out of 12).

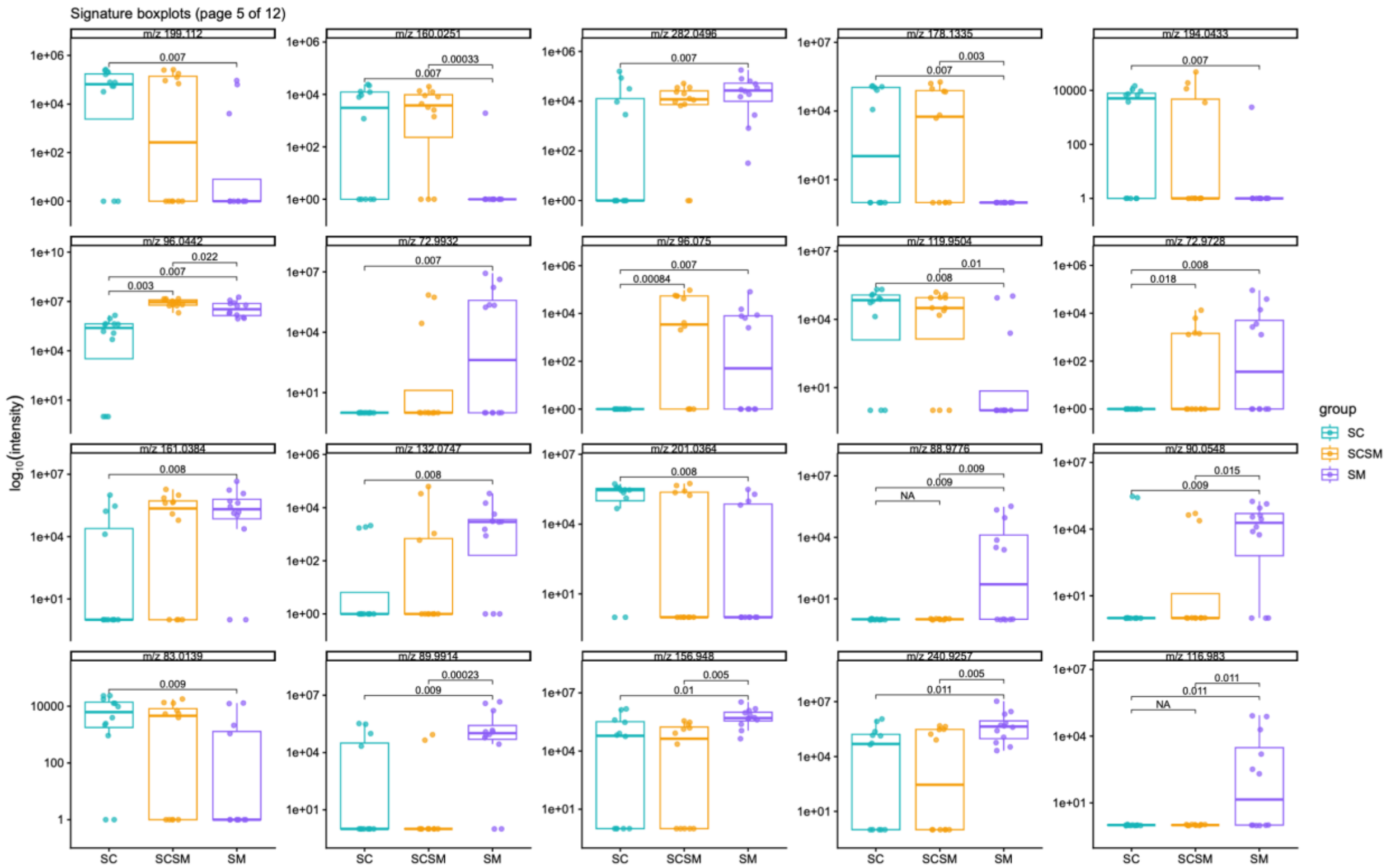

Fig. S16. Boxplots showing the absolute MS intensities in counts per second of the 221 features composing the Serratia signature from the PLS-DA algorithm (part 5 out of 12).

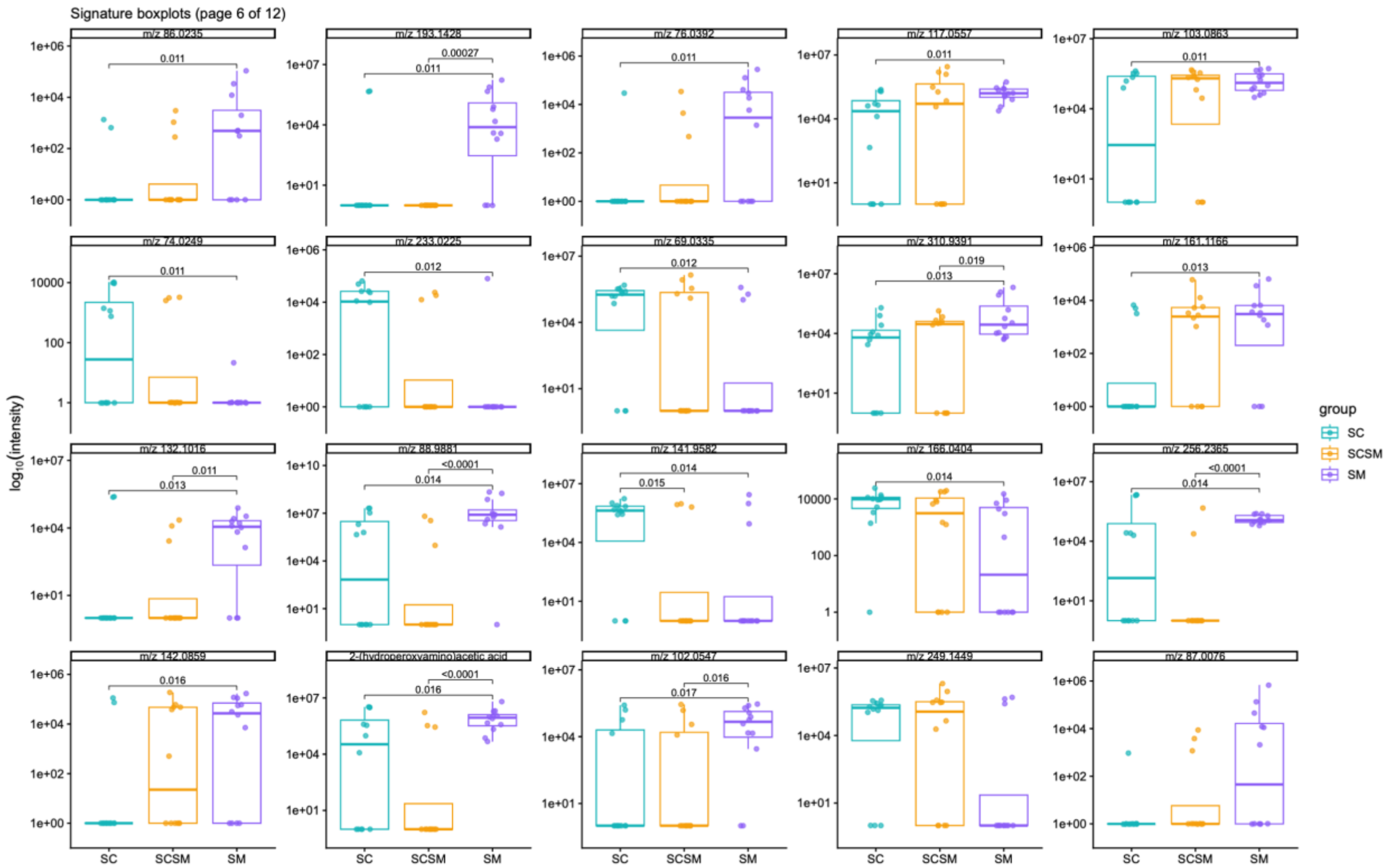

Fig. S17. Boxplots showing the absolute MS intensities in counts per second of the 221 features composing the Serratia signature from the PLS-DA algorithm (part 6 out of 12).

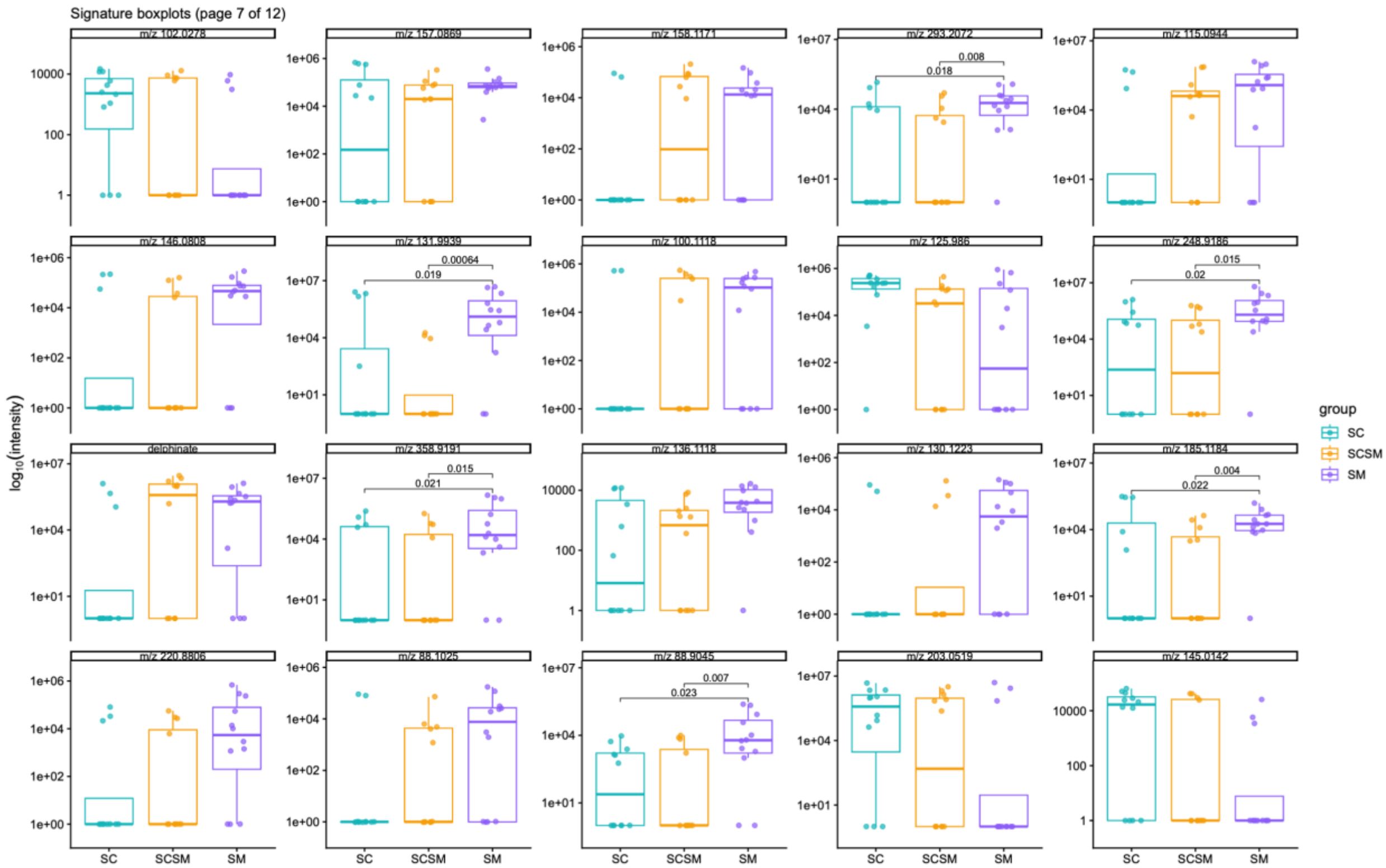

Fig. S18. Boxplots showing the absolute MS intensities in counts per second of the 221 features composing the Serratia signature from the PLS-DA algorithm (part 7 out of 12).

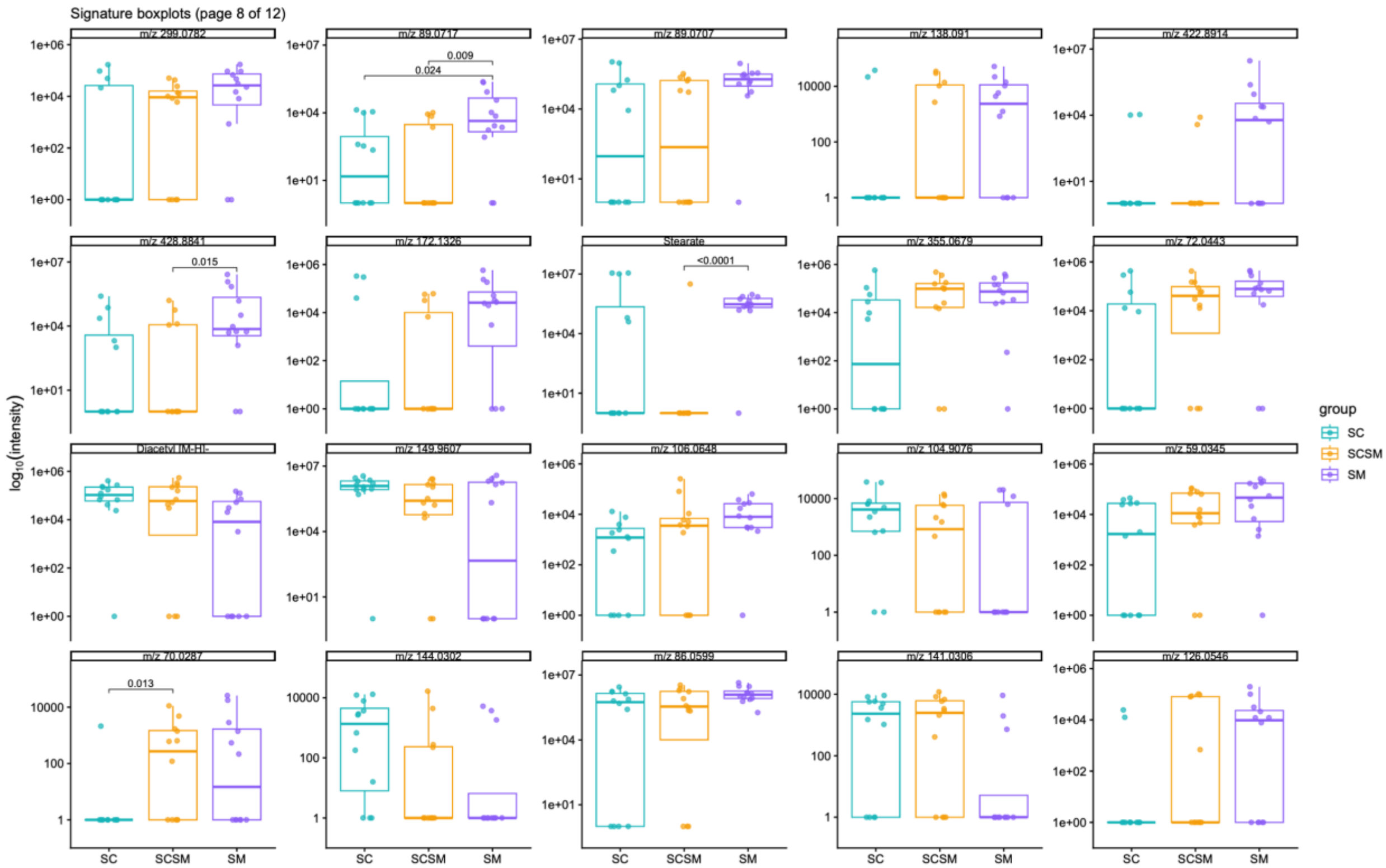

Fig. S19. Boxplots showing the absolute MS intensities in counts per second of the 221 features composing the Serratia signature from the PLS-DA algorithm (part 8 out of 12).

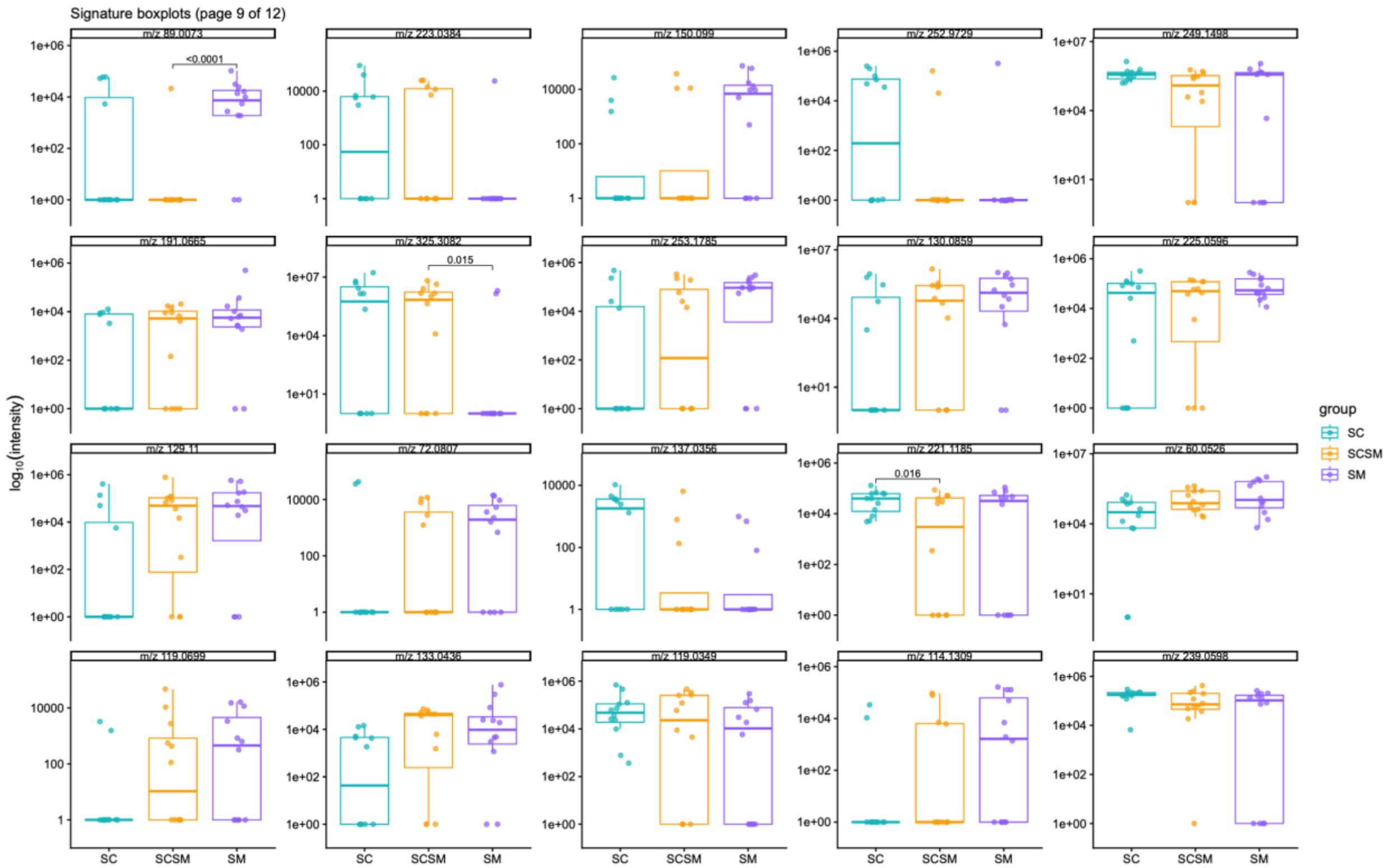

Fig. S20. Boxplots showing the absolute MS intensities in counts per second of the 221 features composing the Serratia signature from the PLS-DA algorithm (part 9 out of 12).

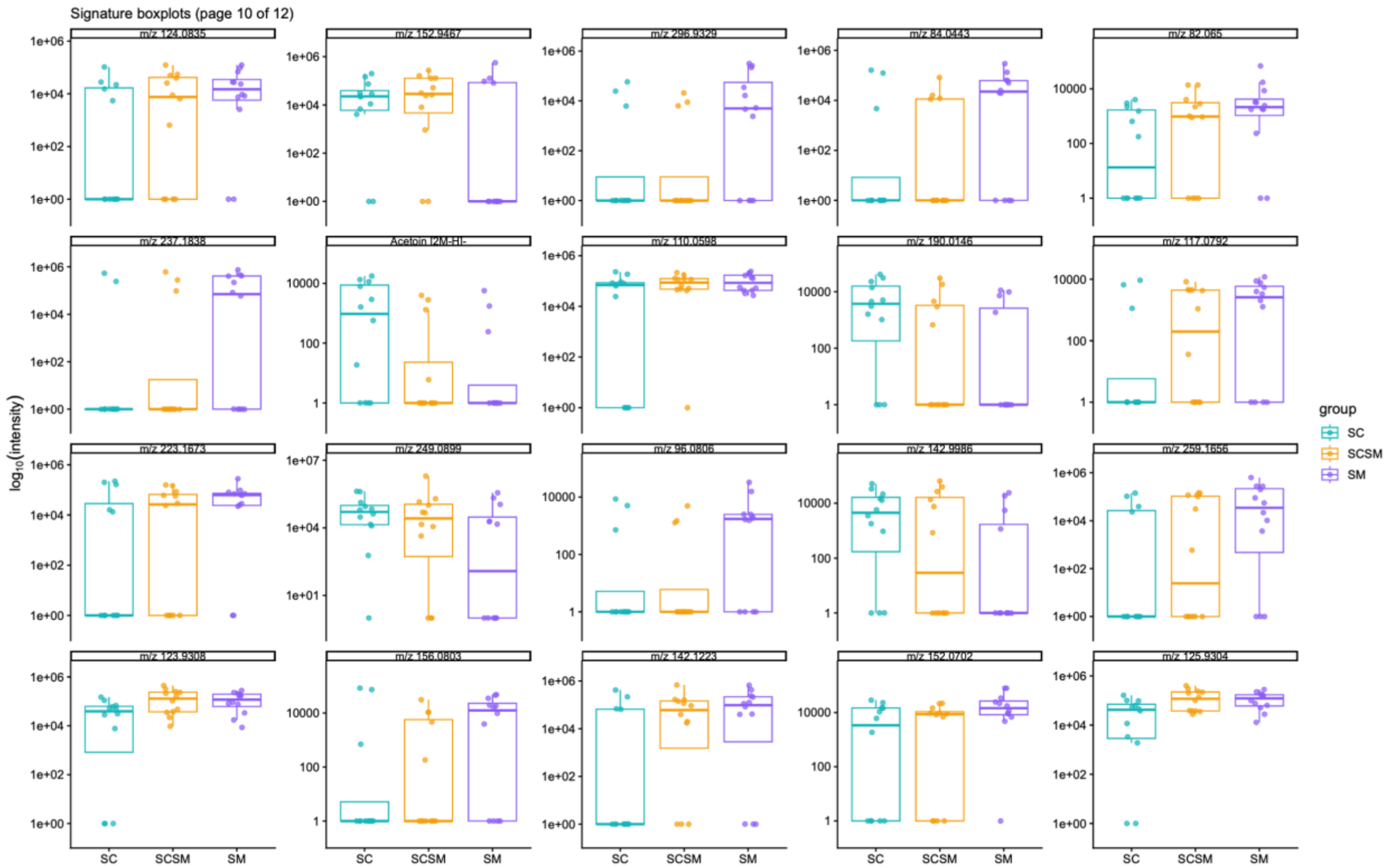

Fig. S21. Boxplots showing the absolute MS intensities in counts per second of the 221 features composing the Serratia signature from the PLS-DA algorithm (part 10 out of 12).

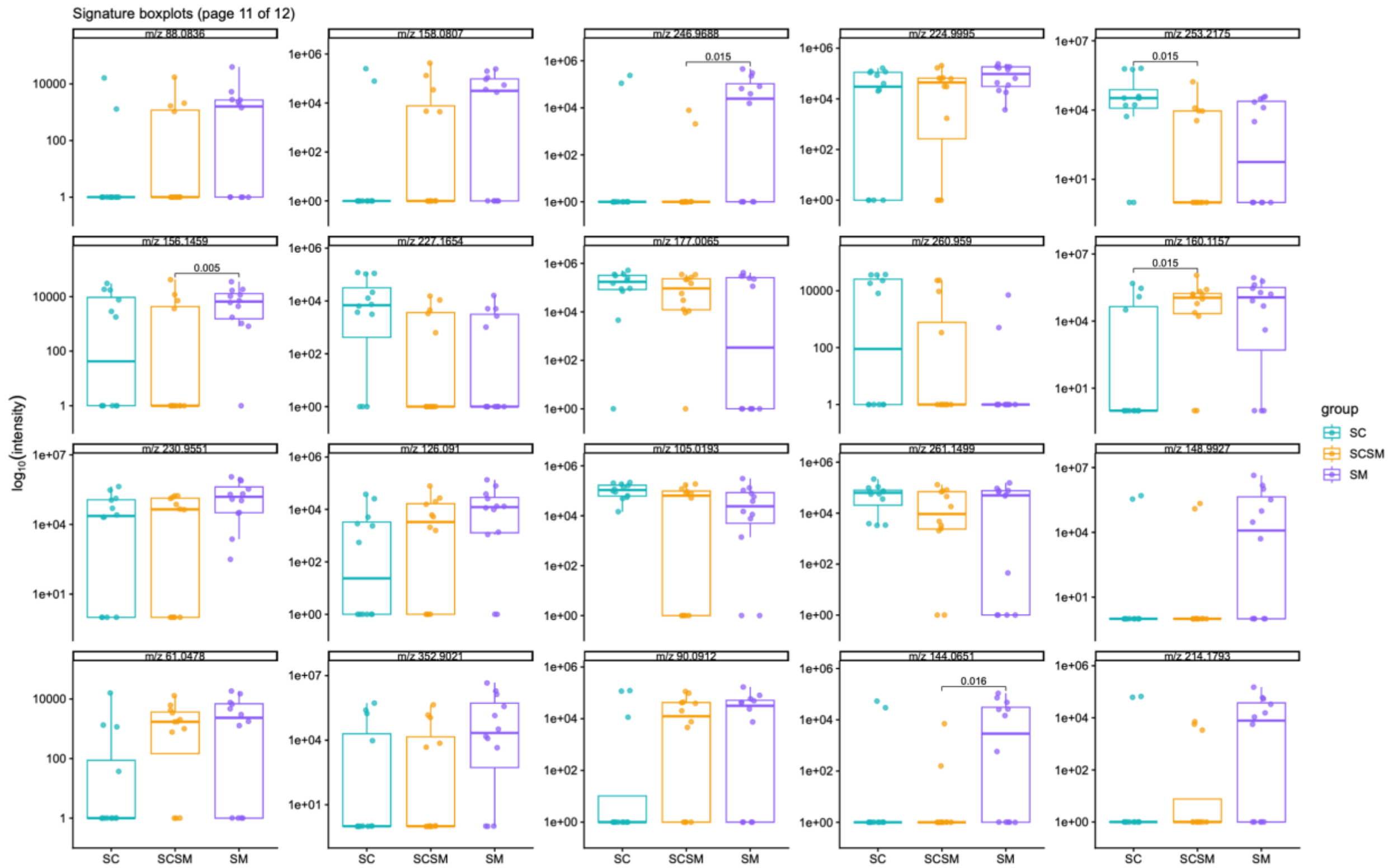

Fig. S22. Boxplots showing the absolute MS intensities in counts per second of the 221 features composing the Serratia signature from the PLS-DA algorithm (part 11 out of 12).

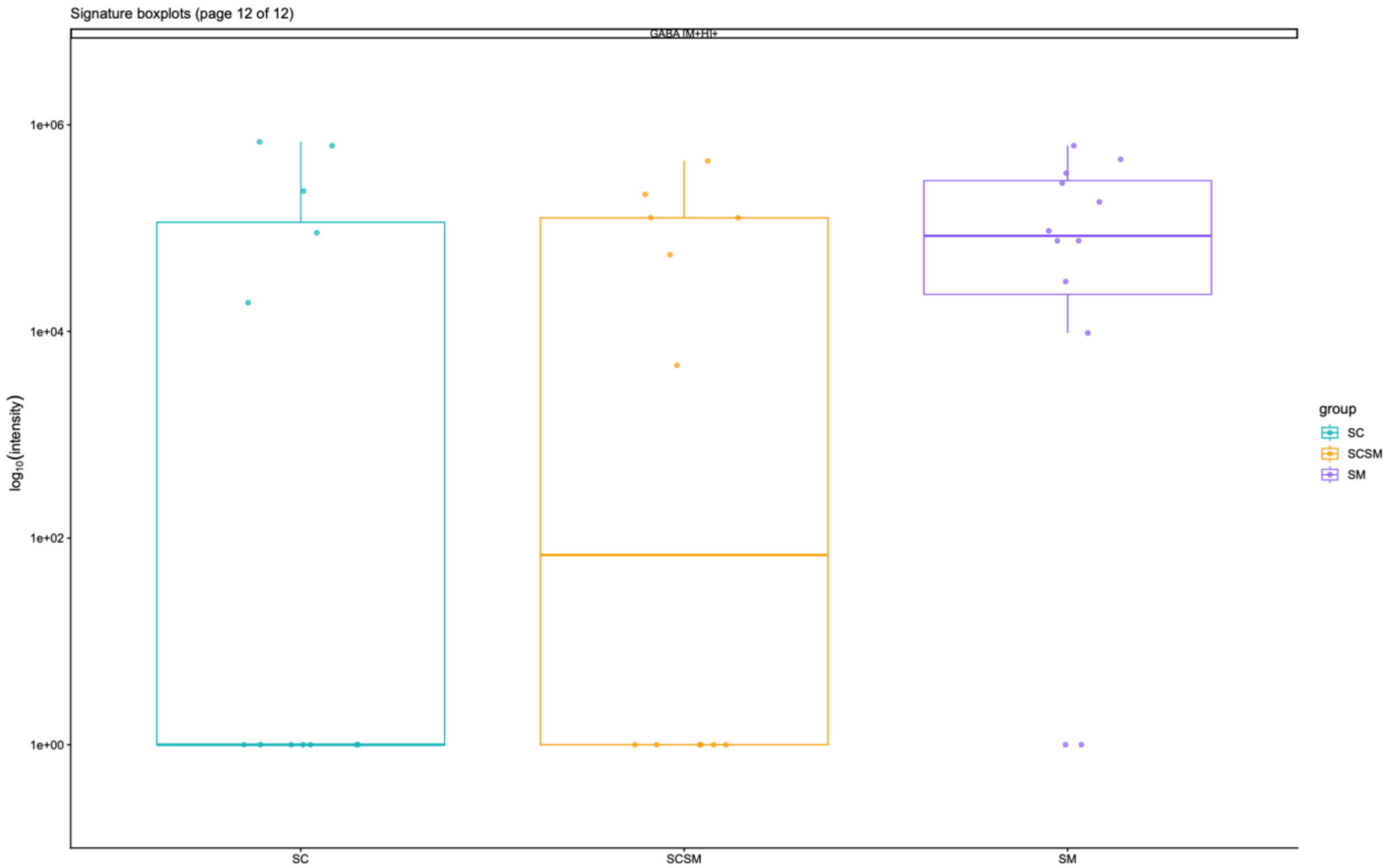

Fig. S23. Boxplots showing the absolute MS intensities in counts per second of the 221 features composing the Serratia signature from the PLS-DA algorithm (part 12 out of 12).

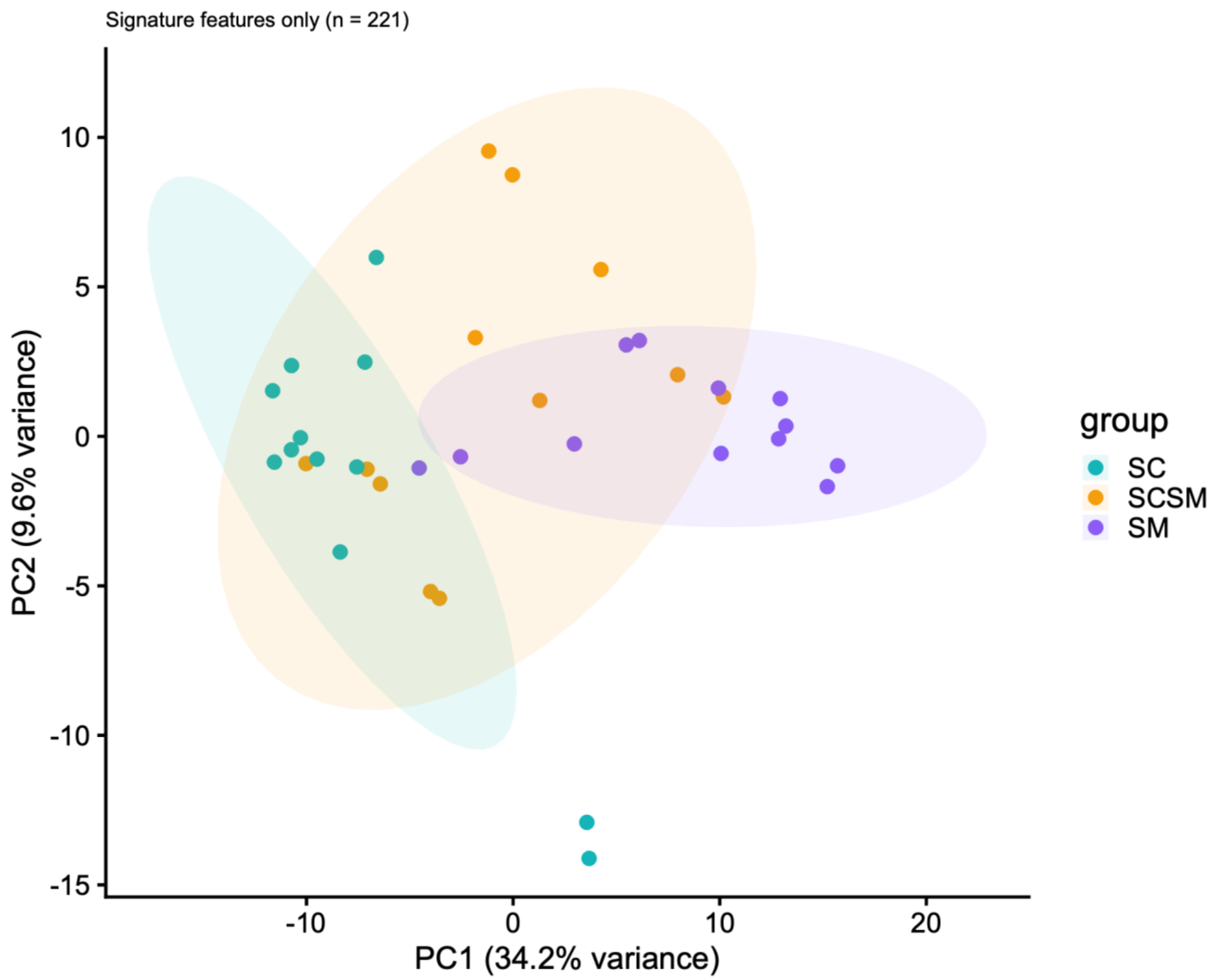

Fig. S24. PCA plot showing the distribution of SC, SCSM, and SM groups when limited to the top 221 features differentiating the SC and SM groups from the PLS-DA distribution.

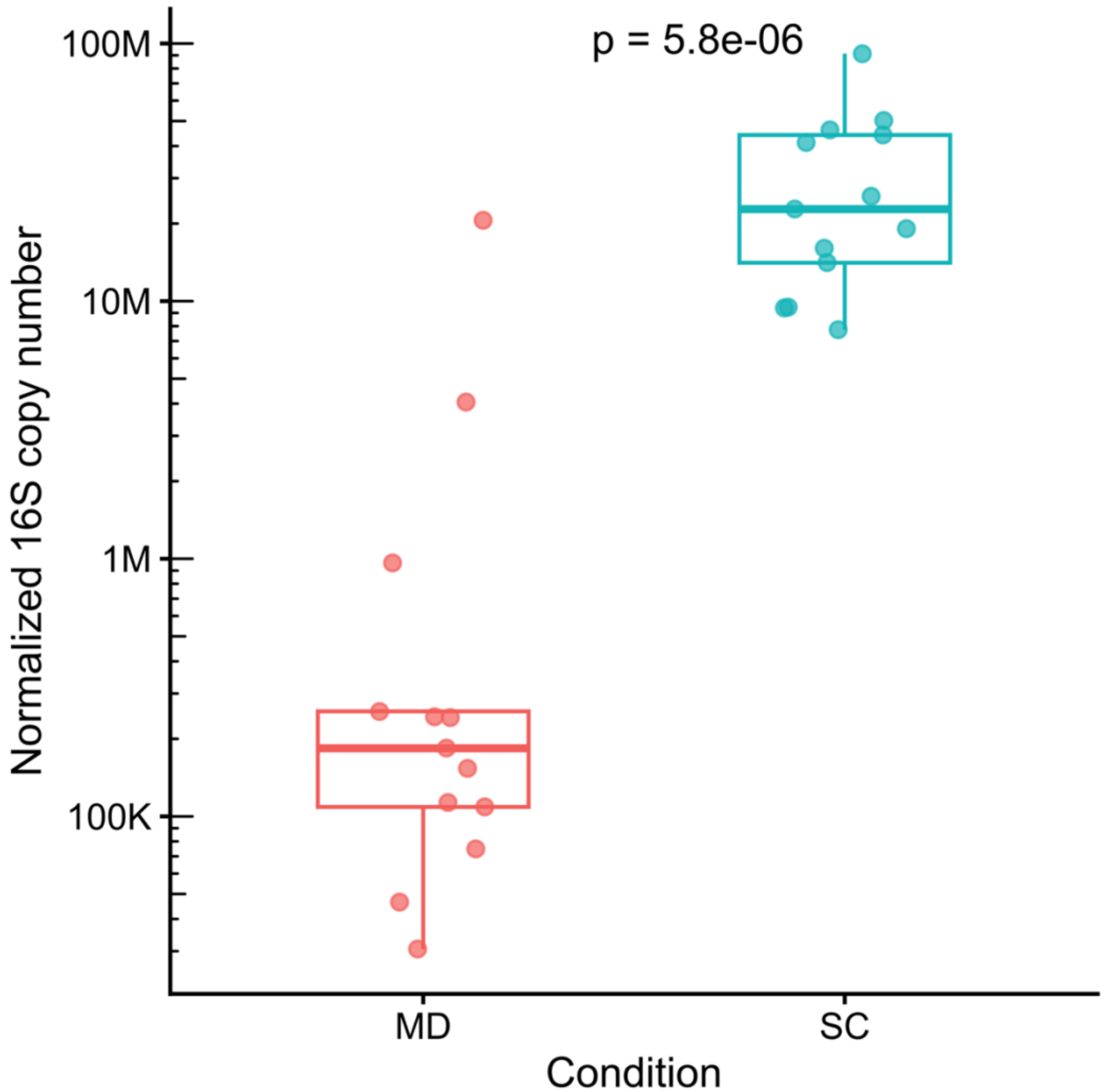

Fig. S25. qPCR bacterial loads across MD and SC gut samples.

Normalized universal 16S rRNA gene copy numbers measured by qPCR in microbiota-depleted (MD) and synthetic-community-colonized (SC) bee gut samples. Individual points are overlaid on boxplots; bacterial loads are normalized to host actin copy number.
